# Supplementary material for: Sensitive red fluorescent indicators for real-time visualization of potassium ion dynamics in vivo
Source: PLoS Biol. 2025 Sep 17;23(9):e3002993. doi: 10.1371/journal.pbio.3002993 (PMC12456824; doi:10.1371/journal.pbio.3002993)
Supplement: S1 Table — (DOCX) [file pbio.3002993.s002.docx]

**S1 Table.** Properties of genetically encoded fluorescent potassium biosensors.

| **Indicators** | | **Ex/Em**  **(nm), apo** | **Quantum Yield (%), apo** | **Extinction Ceofficient (M^-1^·cm^-1^), apo** | **Bright-ness, apo** | **pK_a,apo_/pK_a,sat_** | **ΔF/F**  **(%)** | **K_d_**  **(mM)** | **Ref.** |
| --- | --- | --- | --- | --- | --- | --- | --- | --- | --- |
| **FRET-based sensors** | | | | | | | | | |
| **KIRIN1^FRET^** | **donor** | 433/475 | ND | ND | 104 | 3.2 | 130 | 1.66 | 1 |
|  | **acceptor** | 515/530 | ND | ND | ND | ND |  |  |  |
| **KIRIN1-GR^FRET^** | **donor** | 505/515 | ND | ND | 251 | 6.2 | 20 | 2.56 |  |
|  | **acceptor** | 559/600 | ND | ND | 128 | 5.3 |  |  |  |
| **GEPII^FRET^** | **donor** | 457/475 | ND | ND | ND | ND | 220 | 2.63 | 2 |
|  | **acceptor** | 515/527 | ND | ND |  |  |  |  |  |
| **Single FP-based sensors** | | | | | | | | | |
| **GINKO1** | | 502/514 | 20 | 17,600 | 4,200 | 7.9/7.4 | 29 | 0.42 | 1 |
| **GINKO2** | | 500/515 | 25 | 3,700 | 930 | 7.6/6.8 | 1,400 | 15.3 | 3 |
| **KRaION1** | | 507/519 | 26 | 12,000 | 3,120 | 7.7/7.4 | 302 | 69 | 4 |
| **KRaION2** | | 506/516 | 23 | 9,300 | 2,139 | 7.7/7.1 | 318 | 96 | 4 |
| **RGEPO1** | | 575/599 | 14.6 | 10,712 | 1,567 | 8.54/7.36 | 844 | 14 | This study |
| **RGEPO2** | | 574/599 | 16.2 | 9,680 | 1,563 | 8.54/7.82 | 544 | 59 | This study |

**Supplement References**

1. Shen, Y. *et al* Genetically encoded fluorescent indicators for imaging intracellular potassium ion concentration. *Commun. Biol.* (2019).
2. Bischof, H. *et al.* Novel genetically encoded fluorescent probes enable real-Time detection of potassium in vitro and in vivo. *Nat.Commun*. (2017)
3. Shen, Y. *et al* A sensitive and specific genetically encoded potassium ion biosensor for in vivo applications across the tree of life. *Plos Biology* (2022)
4. Cristina C *et al.* Tuning the Sensitivity of Genetically Encoded Fluorescent Potassium Indicators through Structure-Guided and Genome Mining Strategies. *ACS sensors* (2022)
