# Supplementary material for: Sensitive red fluorescent indicators for real-time visualization of potassium ion dynamics in vivo
Source: PLoS Biol. 2025 Sep 17;23(9):e3002993. doi: 10.1371/journal.pbio.3002993 (PMC12456824; doi:10.1371/journal.pbio.3002993)
Supplement: S2 Table — (DOCX) [file pbio.3002993.s003.docx]

**S2 Table. Photophysical properties of RGEPO1 sensor**

| **Properties** | **free** | **sat** | **X_A_^(sat)^/X_A_^(free)^** | **F_A_^(sat)^/F_A_^(free)^**  **measured directly** |
| --- | --- | --- | --- | --- |
| Relative fraction of “neutral” chromophore (ρ_N_) | 0.915 | 0.68 |  |  |
| Relative fraction of “anionic” chromophore (ρ_A_) | 0.085 | 0.32 | 3.8 |  |
| “Neutral” extinction coefficient (ε_N_, mM^-1^ cm^-1^) ^a^ | 32 | 39 |  |  |
| “Anionic” extinction coefficient (ε_A_, mM^-1^ cm^-1^) ^b^ | 81 | 80 | 0.99 |  |
| “Anionic” Fluorescence lifetime (τ, ns) | 1.98 (57%)  0.50 (43%)  <t> = 1.35 | 1.74 (63%)  0.63 (37%)  <t> = 1.33 |  |  |
| “Anionic” quantum yield (ϕ_A_) | 0.14* | 0.18* | 1.29 |  |
| “Neutral” quantum yield (ϕ_N_) | N/A | N/A |  |  |
| “Anionic” molecular brightness (ρ_A_ x ε_A_ x ϕ_A_) | 0.96 | 4.6 | 4.8 | 4.7 |
| “Neutral” 1PA peak (nm) | 446 | 446 |  |  |
| “Anionic” 1PA peak (nm) | 574 | 564 |  |  |
| “Anionic” two-photon cross section (GM at λ, nm) | 27  (1060) | 40  (1060) | 1.48 |  |
| “Anionic” two-photon brightness, F_2_ (GM at λ, nm) | 0.33  (1068 nm) | 2.3  (1060) | 7.0 | 8.5  (1060nm) |

*Measured vs cresyl violet in methanol.

^a,b^Here we define ε_A_ and ε_N_ as an optical density of 1-molar concentration of either anionic or neutral form of chromophore in 1-cm cuvette.
