# Supplementary material for: Sensitive red fluorescent indicators for real-time visualization of potassium ion dynamics in vivo
Source: PLoS Biol. 2025 Sep 17;23(9):e3002993. doi: 10.1371/journal.pbio.3002993 (PMC12456824; doi:10.1371/journal.pbio.3002993)
Supplement: S3 Table — (DOCX) [file pbio.3002993.s004.docx]

**S3 Table. Photophysical properties of RGEPO2 sensor**

| **Properties** | **free** | **sat** | **X_A_^(sat)^/X_A_^(free)^** | **F_A_^(sat)^/F_A_^(free)^**  **measured directly** |
| --- | --- | --- | --- | --- |
| Relative fraction of “neutral” chromophore (ρ_N_) | 0.89 | 0.70 |  |  |
| Relative fraction of “anionic” chromophore (ρ_A_) | 0.11 | 0.30 | 2.7 |  |
| “Neutral” extinction coefficient (ε_N_, mM^-1^ cm^-1^) ^a^ | 38 | 33.5 |  |  |
| “Anionic” extinction coefficient (ε_A_, mM^-1^ cm^-1^) ^b^ | 74 | 69 | 0.93 |  |
| “Anionic” Fluorescence lifetime (τ, ns) | 1.75 (54%)  0.28 (46%)  <t> = 1.07 | 1.57 (62%)  0.25 (38%)  <t> = 1.07 |  |  |
| “Anionic” quantum yield (ϕ_A_) | 0.12* | 0.20* | 1.67 |  |
| “Neutral” quantum yield (ϕ_N_) | N/A | N/A |  |  |
| “Anionic” molecular brightness (ρ_A_ x ε_A_ x ϕ_A_) | 0.98 | 4.1 | 4.2 | 4.3  (564nm) |
| “Neutral” 1PA peak (nm) | 446 | 446 |  |  |
| “Anionic” 1PA peak (nm) | 576 | 564 |  |  |
| “Anionic” two-photon cross section (GM at λ, nm) | 22  (1060) | 33  (1060) | 1.50 |  |
| “Anionic” two-photon brightness, F_2_ (GM at λ, nm) | 0.30  (1068 nm) | 2.0  (1060) | 6.7 | 6.4  (1060nm) |

*****Measured vs cresyl violet in methanol.

^a,b^ Here we define ε_A_ and ε_N_ as an optical density of 1-molar concentration of either anionic or neutral form of chromophore in 1-cm cuvette.
