# Supplementary figures and images for: Sensitive red fluorescent indicators for real-time visualization of potassium ion dynamics in vivo
Source: PLoS Biol. 2025 Sep 17;23(9):e3002993. doi: 10.1371/journal.pbio.3002993 (PMC12456824; doi:10.1371/journal.pbio.3002993)

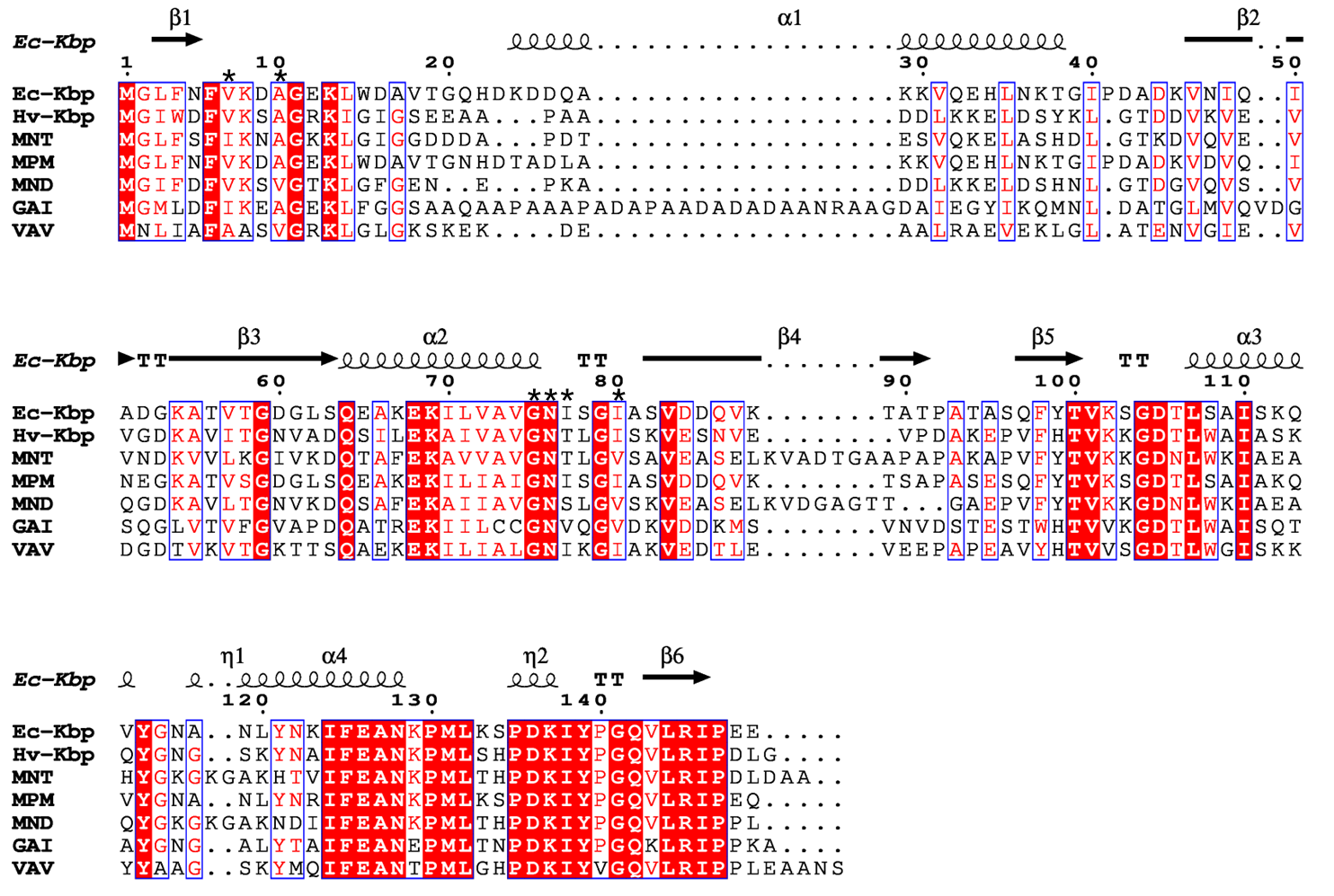

Supplement: S1 Fig — Residues within the red boxes with white text indicate the strict identity of amino acids, while those within a blue frame denote similarity among the homologs. Labels for β-sheet-forming regions and α-helix-forming regions are provided as arrows/straight lines and curved lines, respectively. The symbol ‘η’ denotes a 310 helix, and ‘TT’ indicates strict β-turns. Residues located within 3.0 Å of the potassium ion in the Ec-Kbp structure (PDB: 7PVC) are marked with asterisks (N = 6 amino acids). The amino acid numbering follows that of Ec-Kbp. Alignment of protein sequence was conducted via Clustal Omega and subsequently shown with Ec-Kbp structure by ESPript 3.0. (TIFF) [file pbio.3002993.s005.tiff]

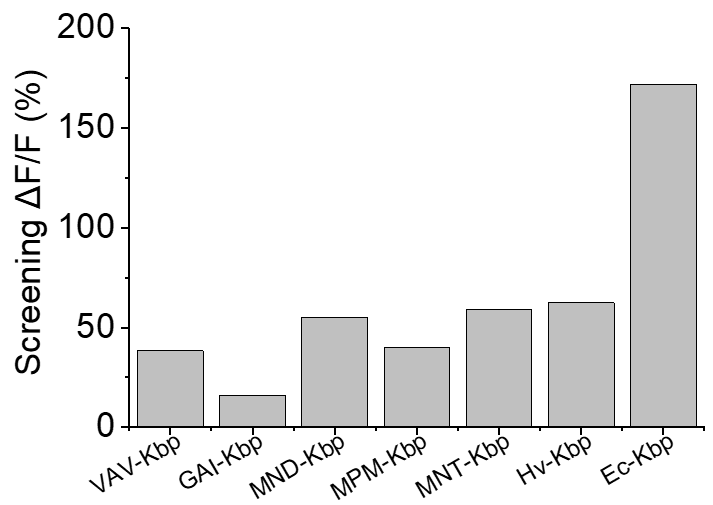

Supplement: S2 Fig — The homologs were inserted into mNeoGreen following the previous KRaION1’s design [4] and titrated with 230 mM K+ in solution. The underlying numerical data for this figure can be found in S1 Data. (TIFF) [file pbio.3002993.s006.tiff]

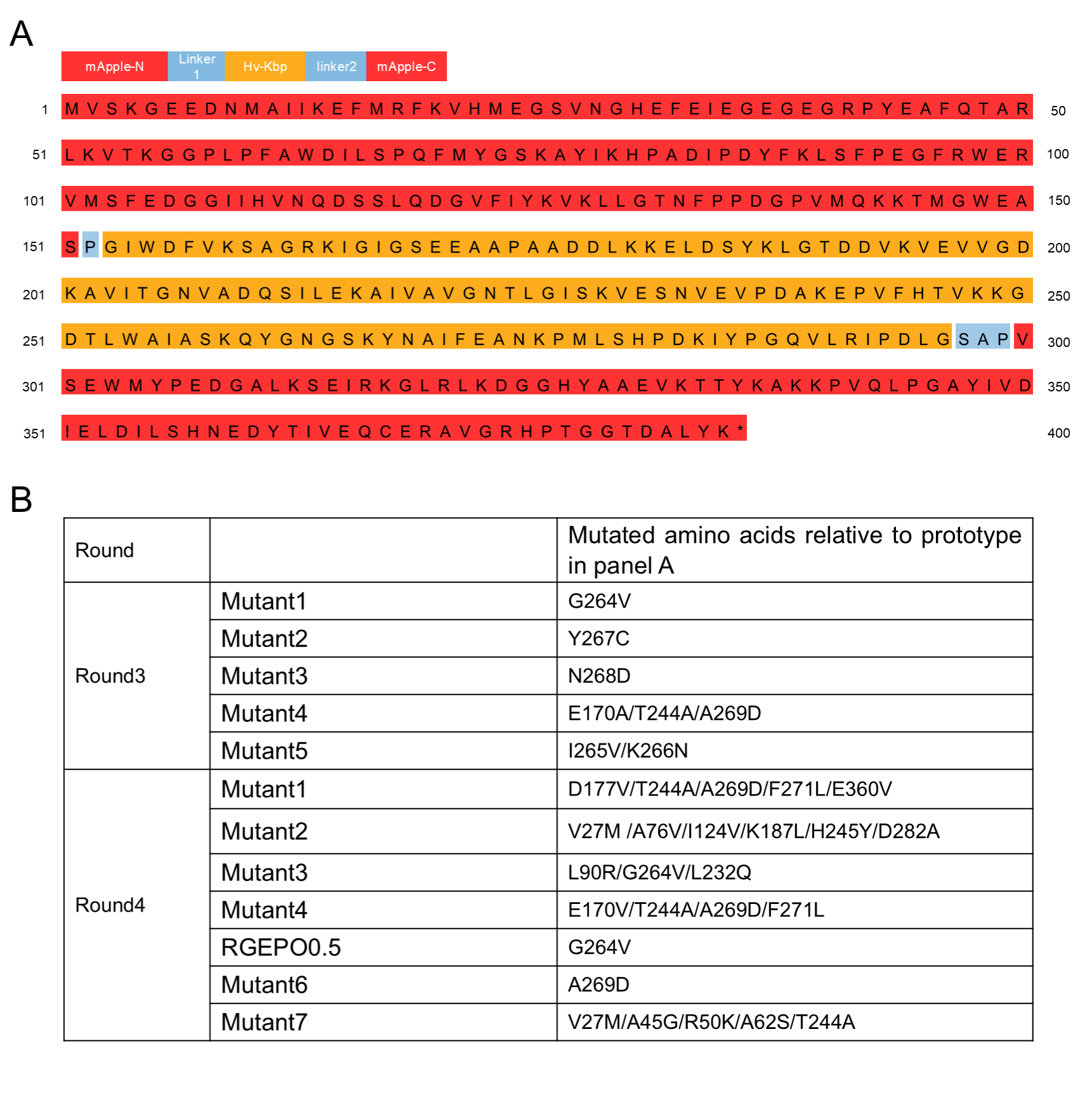

Supplement: S3 Fig — (A) Amino acids sequence of RGEPO-prototype sensor with linker sequences Linker1: P and Linker2: SAP. (B) The mutants were validated in mammalian cells, corresponding to the red-highlighted dots in Fig 1E. (TIFF) [file pbio.3002993.s007.tiff]

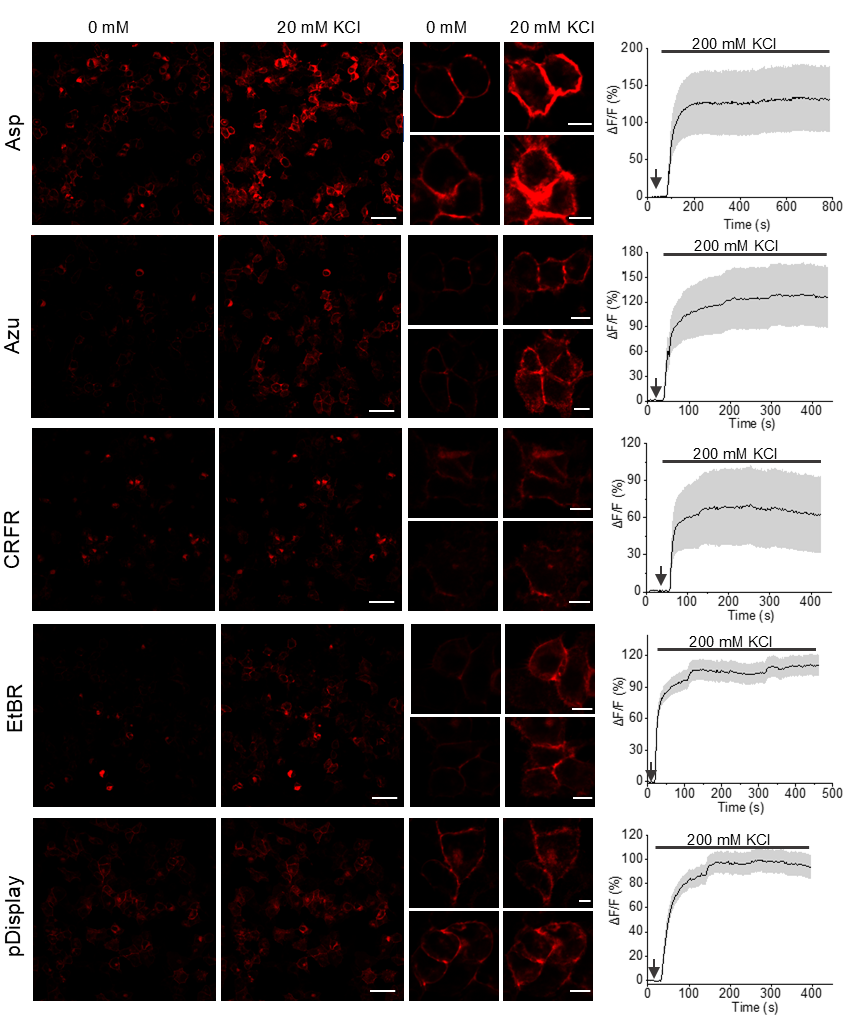

Supplement: S4 Fig — Left, single plane fluorescence images of RGEPOs with five different leader sequences expressed in HEK293FT cells in response 20 mM KCl (n = 2 field of views (FOVs) from 1 culture). Scale bars, 50 µm. Middle, magnified images in K+ free and K+ binding state (n = 3 cells from 2 FOVs over 1 culture for each). Scale bars, 10 µm. Right, single-trial optical traces of RGEPOs with different leader sequence upon stimulation of 200 mM KCl, which is shown in the Fig 2E (n = 31, 13, 22, 22, 24 cells from 1 culture for Asp, Azu, CRFR, EtbR, and Igκ, respectively). The underlying numerical data for this figure can be found in S1 Data. (TIFF) [file pbio.3002993.s008.tiff]

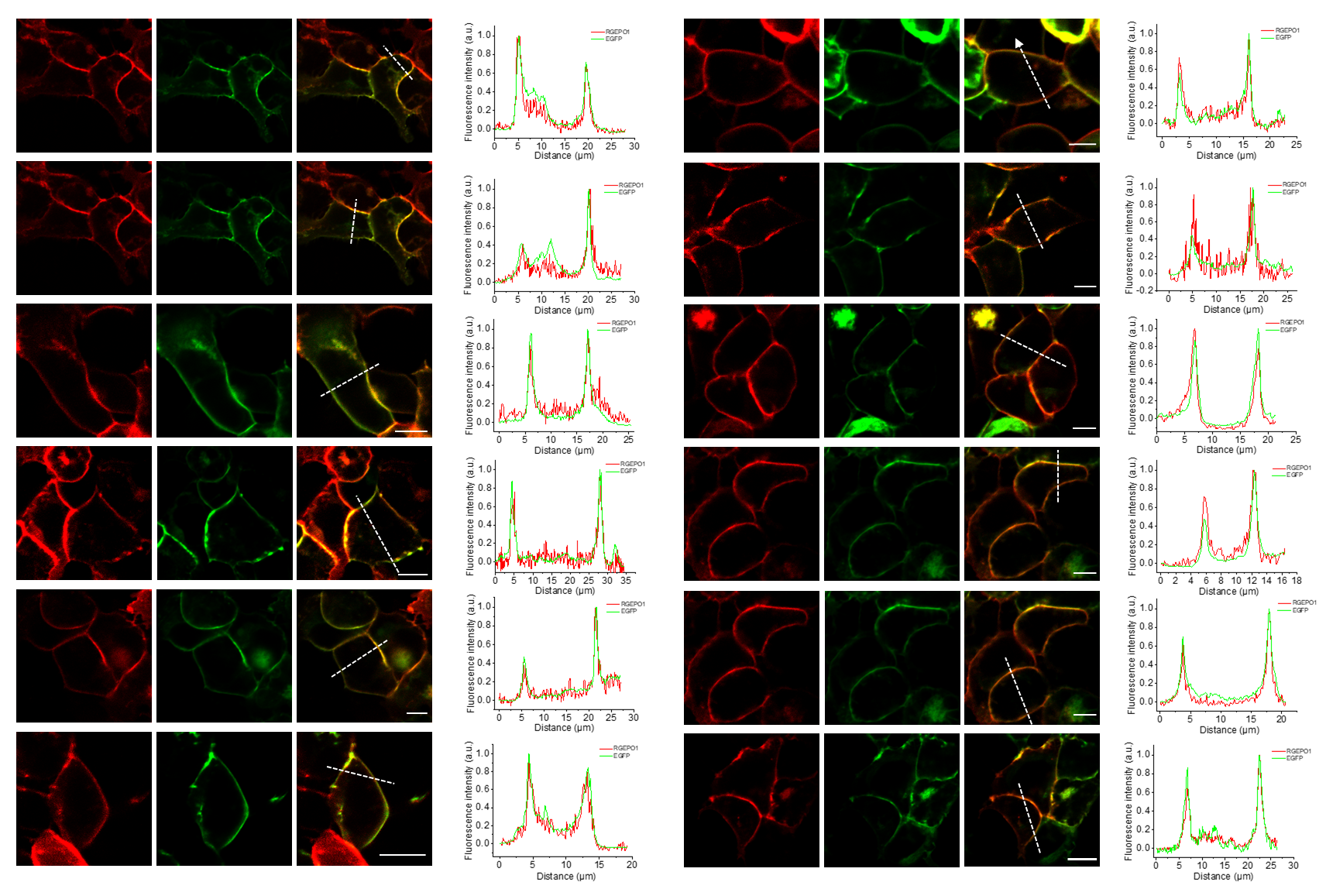

Supplement: S5 Fig — Fluorescence images and membrane localization analysis of Asp-RGEPO0.5 expressed in HEK293FT cells. Membrane-targeted GFP (Igκ-GFP) was co-expressed to label the plasma membrane. Left, single-plane confocal fluorescence images of HEK293T cells expressing the RGEPO0.5 (red) and EGFP (green) (n = 12 cells from 2 independent transfections from one culture). Right, normalized linecut (shown as white dashed line on the left) plots of fluorescence signals measured in both the red and green channels. Scale bars, 10 µm. The underlying numerical data for this figure can be found in S1 Data. (TIFF) [file pbio.3002993.s009.tiff]

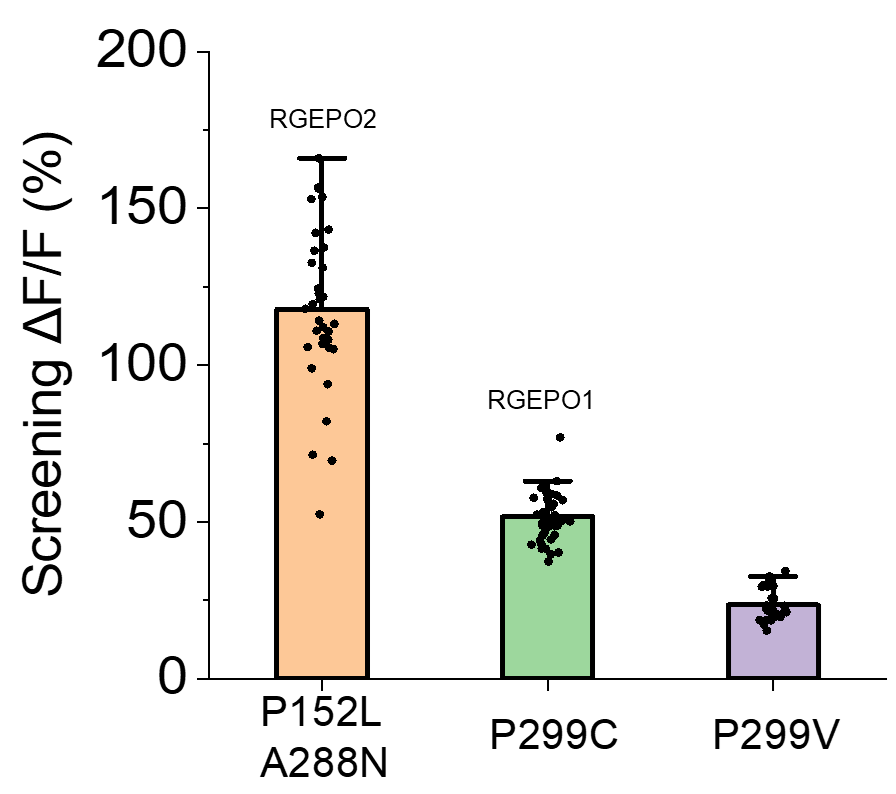

Supplement: S6 Fig — Validation of the top three mutants, which exhibited the highest extracellular responses, in the intracellular environment using a perfusion system (n = 34, 44, 33 cells from 1 culture each, respectively). Dot, individual data point for single cells; bar, mean; error bar, SD. The underlying numerical data for this figure can be found in S1 Data. (TIFF) [file pbio.3002993.s010.tiff]

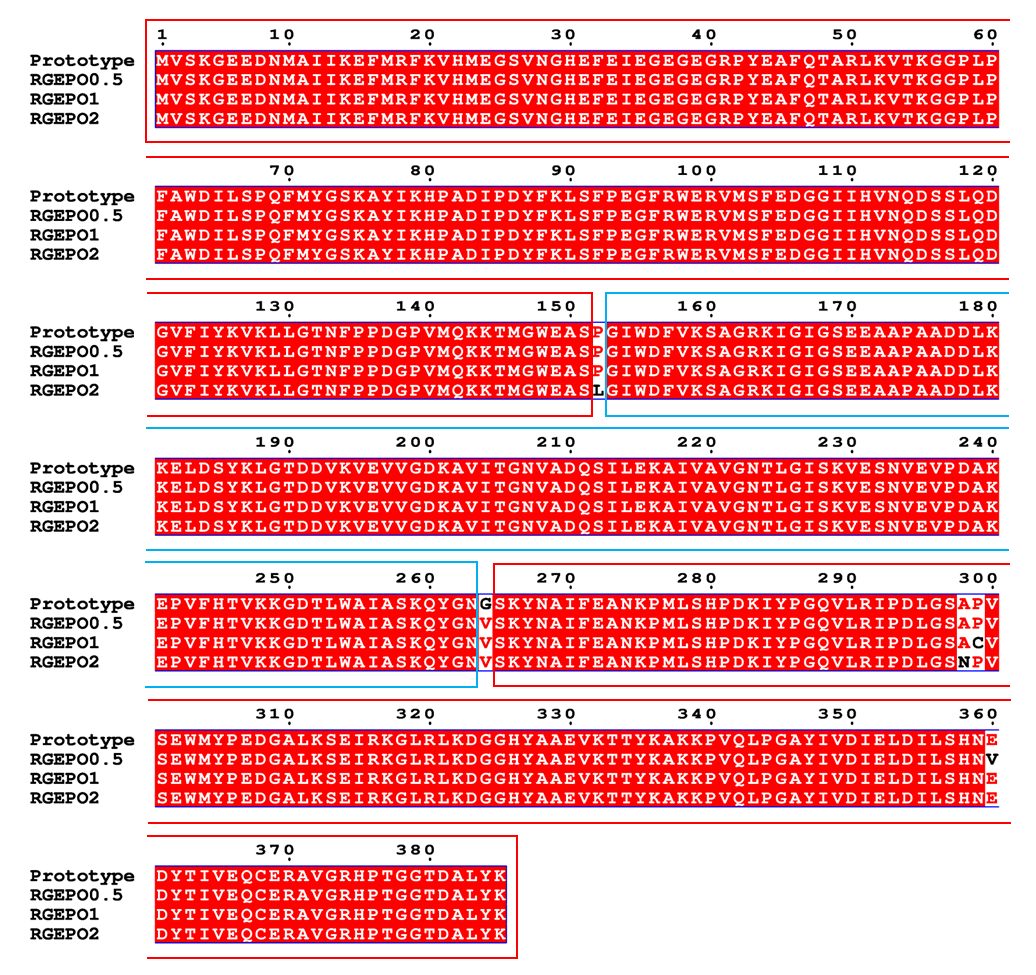

Supplement: S7 Fig — The sequences derived from mApple and the potassium-binding domain are highlighted in red and blue boxes, respectively. The nonred shaded areas indicate mutations acquired during the engineering process. (TIFF) [file pbio.3002993.s011.tiff]

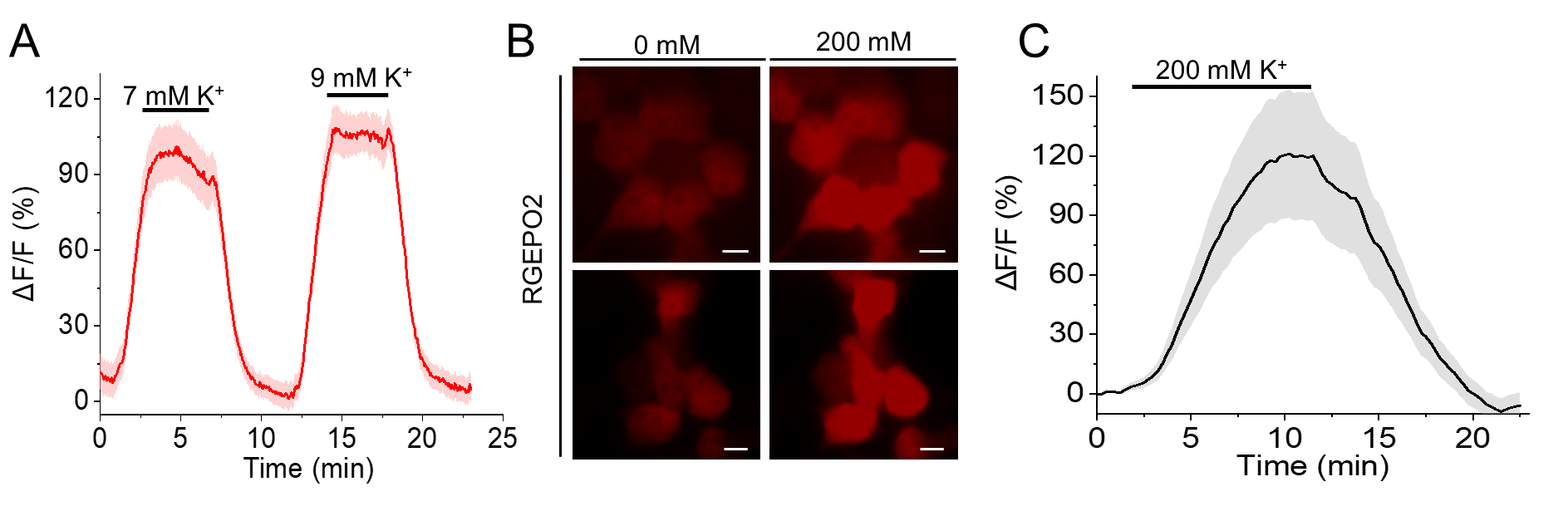

Supplement: S8 Fig — (A) Fluorescence intensity change (ΔF/F0) time course of RGEPO1 with stimulation by a series of K+ buffer on HEK293T cells (n = 5 cells from 1 culture), data are expressed as mean (solid line) and SD (shaded area). (B) Representative images of RGEPO2 expressed in the HEK293FT cells (n = 32 cells from 1 cultures). Scale bar, 10 µm. Images were obtained via an inverted wide-field Nikon Eclipse Ti2 microscope with 20× NA0.75 objective lens. (C) Fluorescence intensity change (ΔF/F) time course of RGEPO2 with stimulation by a series of K+ buffer on HEK293T cells using valinomycin and CCCP (n = 34 cells from 1 culture), data are expressed as mean (solid line) and SD (shaded area). The underlying numerical data for this figure can be found in S1 Data. (TIFF) [file pbio.3002993.s012.tiff]

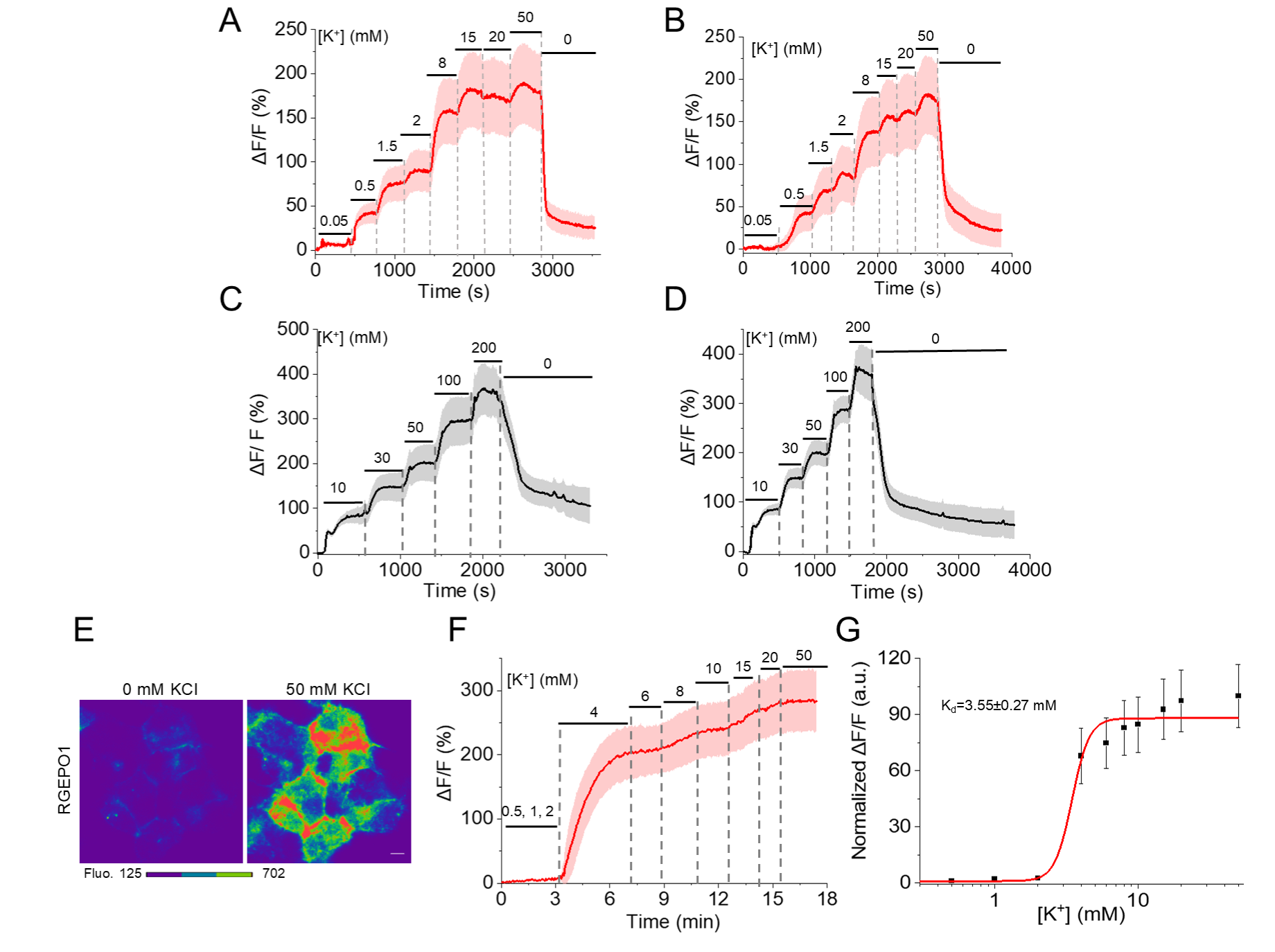

Supplement: S9 Fig — (A, B) Time courses of fluorescence intensity change (ΔF/F) of RGEPO1 on HEK293FT cells stimulated with a series of K⁺ buffers. Each trace represents data from an independent culture (n = 33 and 25 cells, respectively). Data are shown as mean ± SD. (C–E) Time courses of fluorescence intensity change (ΔF/F) of RGEPO2 in HEK cells stimulated with a series of K⁺ buffers in the presence of 10 μg/mL gramicidin. Each trace represents data from an independent culture (n = 32 and 36 cells, respectively). Data are shown as mean ± SD. (F) Representative images of expression and fluorescence change of RGEPO1 in response to 20 mM K+. Scale bar, 10 µm. (G) Fluorescence intensity change (ΔF/F) time course of RGEPO1 with stimulation by a series of K+ buffers on HEK293T cells (n = 18 cells from 1 culture), data are expressed as mean ± SD. The titration was performed by manual addition of each buffer. (H) Plot of normalized ∆F/F against different K+ concentrations fitted using nonlinear fitting (Hill) for the data shown in panel G. The underlying numerical data for this figure can be found in S1 Data. (TIFF) [file pbio.3002993.s013.tiff]

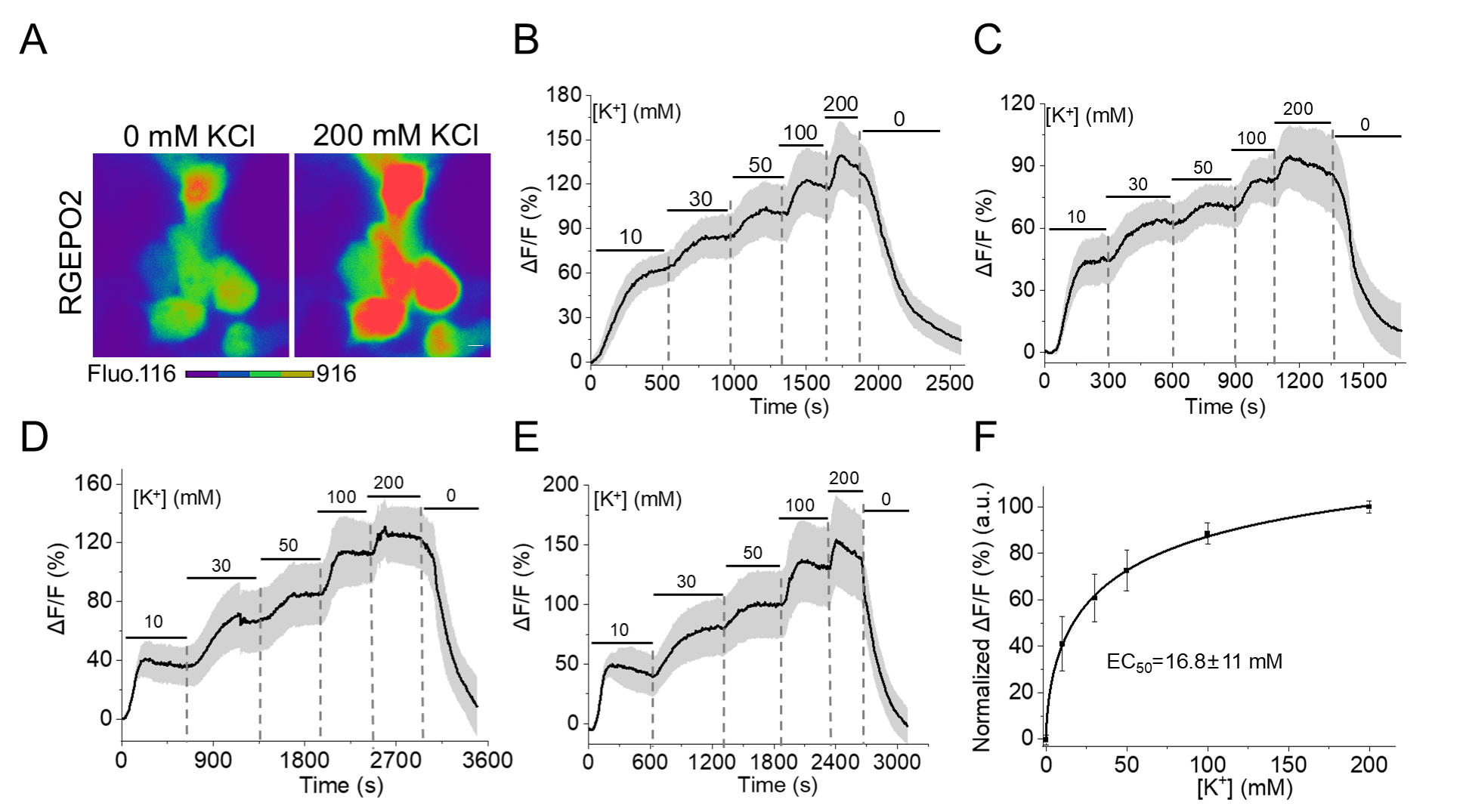

Supplement: S10 Fig — (A) Representative images of expression and fluorescence change of RGEPO2 in response to 200 mM K+. Scale bar, 10 µm. (B–E) Time courses of fluorescence intensity change (ΔF/F) of RGEPO2 in HEK cells stimulated with a series of K⁺ buffers in the presence of valinomycin and CCCP. Trace represents data from 3 independent culture (n = 32, 22, 10 and 16 cells, respectively). Data are shown as mean ± SD. (F) Plot of normalized ∆F/F against different K+ concentrations fitted using nonlinear fitting (Hill) for the data shown in panels C, D,and E (n = 80 cells from 3 independent cultures). The underlying numerical data for this figure can be found in S1 Data. (TIFF) [file pbio.3002993.s014.tiff]

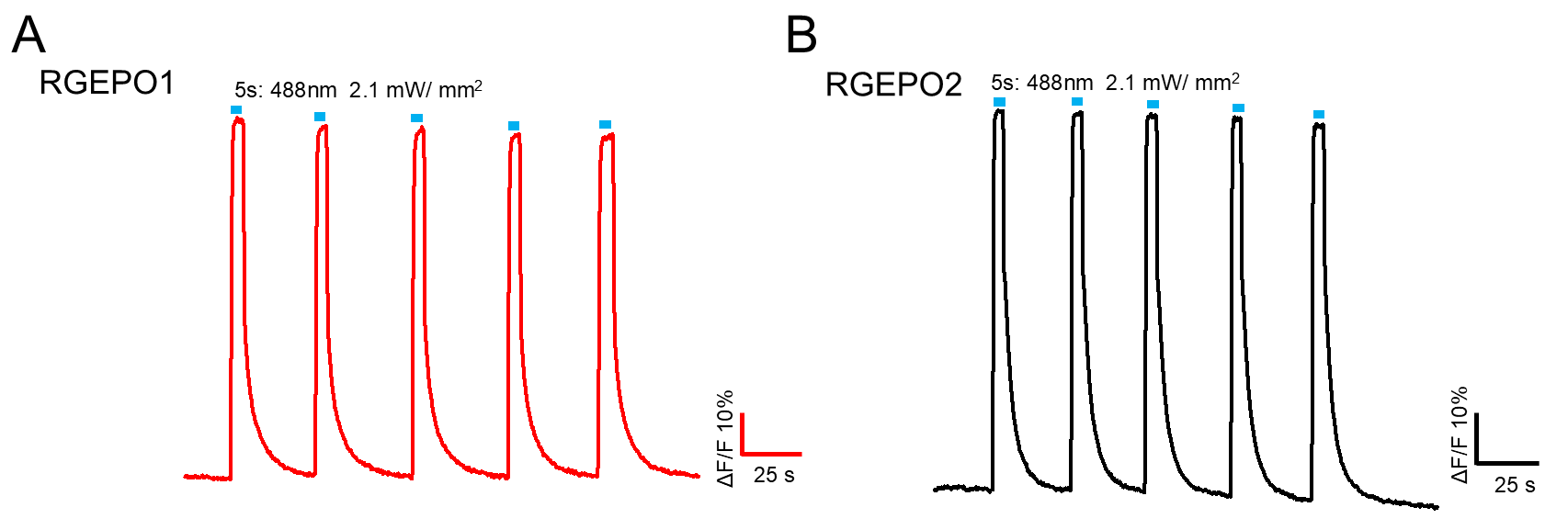

Supplement: S11 Fig — Photoactivation of RGEPO1 (n = 10 cells) and RGEPO2 (n = 8 cells) was assessed in HEK293FT cells by continuous imaging with green-light excitation (555/20 nm), combined with five additional blue-light stimulations (470/28 nm, 2.1 mW/ mm2, 5 s pulse, as indicated). The underlying numerical data for this figure can be found in S1 Data. (TIFF) [file pbio.3002993.s015.tiff]

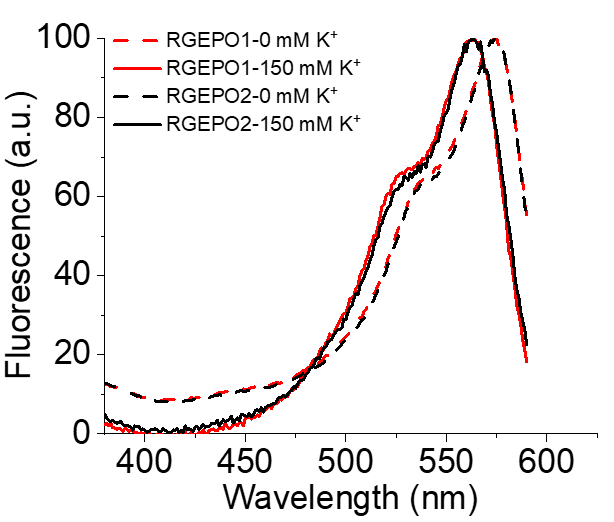

Supplement: S12 Fig — The underlying numerical data for this figure can be found in S1 Data. (TIFF) [file pbio.3002993.s016.tiff]

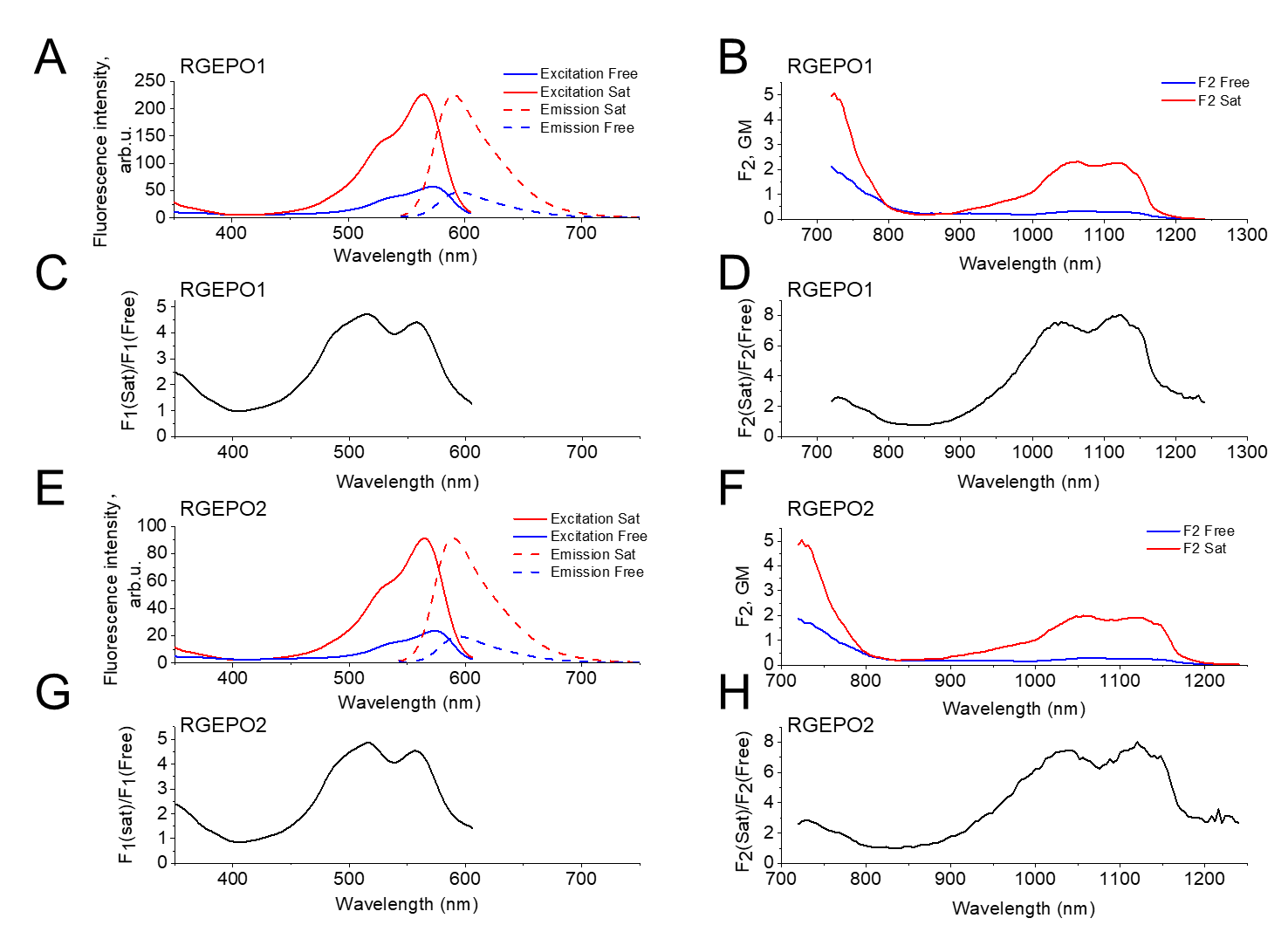

Supplement: S13 Fig — Fluorescence emission and excitation spectra of K⁺-free (blue lines) and K⁺-saturated (red lines) states of RGEPO1: (A, B) and RGEPO2: (E–F). Left side of the figure (panels A, C, E, G) corresponds to spectra obtained under one-photon excitation. Excitation spectra are shown as solid lines, and emission spectra as dashed lines: (A) and (E). Panels (C) and (D) represent the ratio of fluorescence intensities of the saturated state versus free state as a function of laser excitation wavelength. Right side of the figure (panels B, D, F, H) corresponds to the two-photon excitation spectra. Panels (D) and (H) represent the ratio of fluorescence intensities of saturated versus free states as a function of two-photon laser excitation wavelength. The underlying numerical data for this figure can be found in S1 Data. The underlying numerical data for this figure can be found in S1 Data. (TIFF) [file pbio.3002993.s017.tiff]

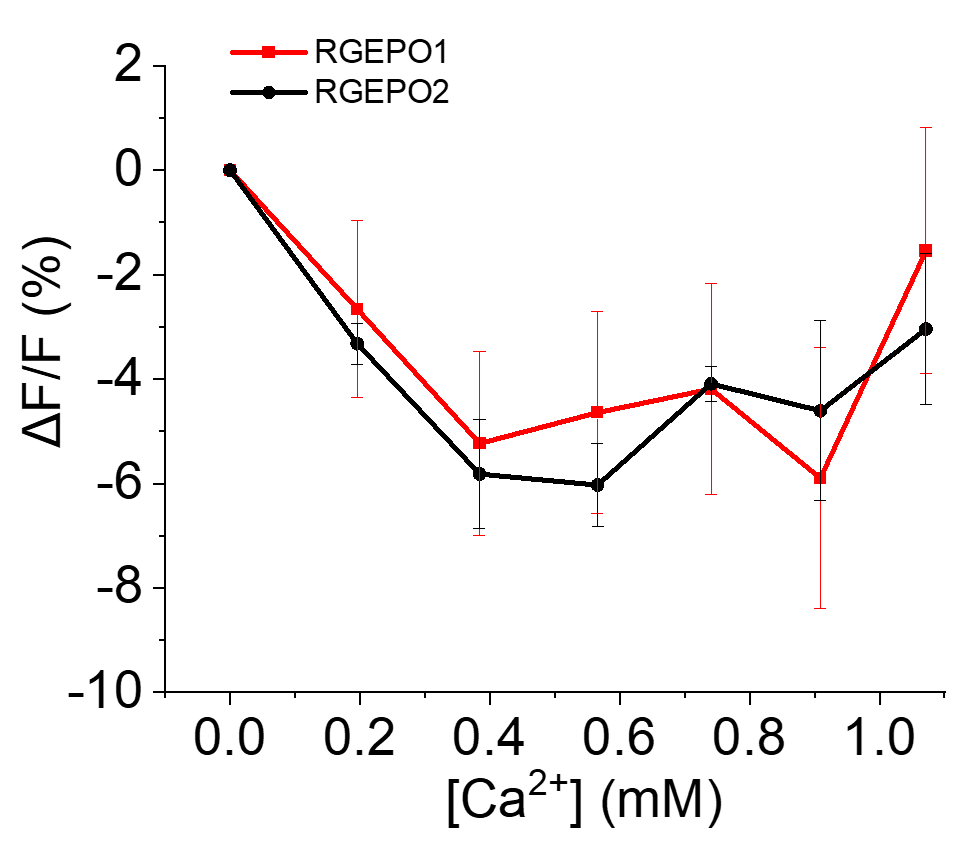

Supplement: S14 Fig — Ca2+ titration of RGEPOs in the range of 0–1.07 mM in solution (n = 3 technical replicates; mean ± SD). The underlying numerical data for this figure can be found in S1 Data. (TIFF) [file pbio.3002993.s018.tiff]

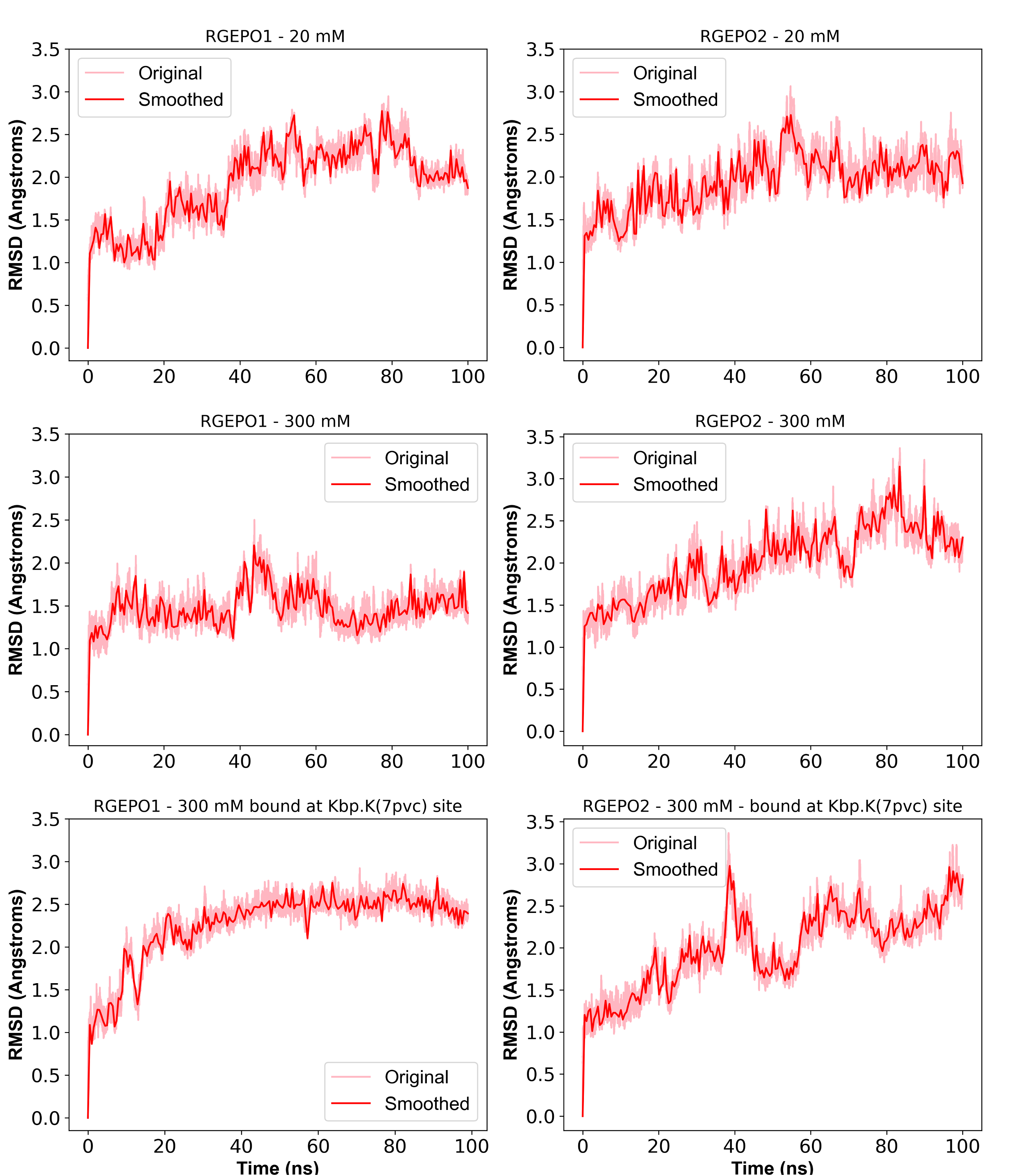

Supplement: S15 Fig — The first two row graphs show the free diffusion simulations of RGEPO1 (left column) and RGEPO2 (right column) at their respective ionic concentrations. The last row graphs represent simulations of RGEPO1 (left) and RGEPO2 (right) with a 300 mM ionic concentration, initiated with a K+ bound at the originally reported Kbp.K binding site (PDB ID: 7PVC). The underlying numerical data for this figure can be found in S1 Data. (TIFF) [file pbio.3002993.s019.tiff]

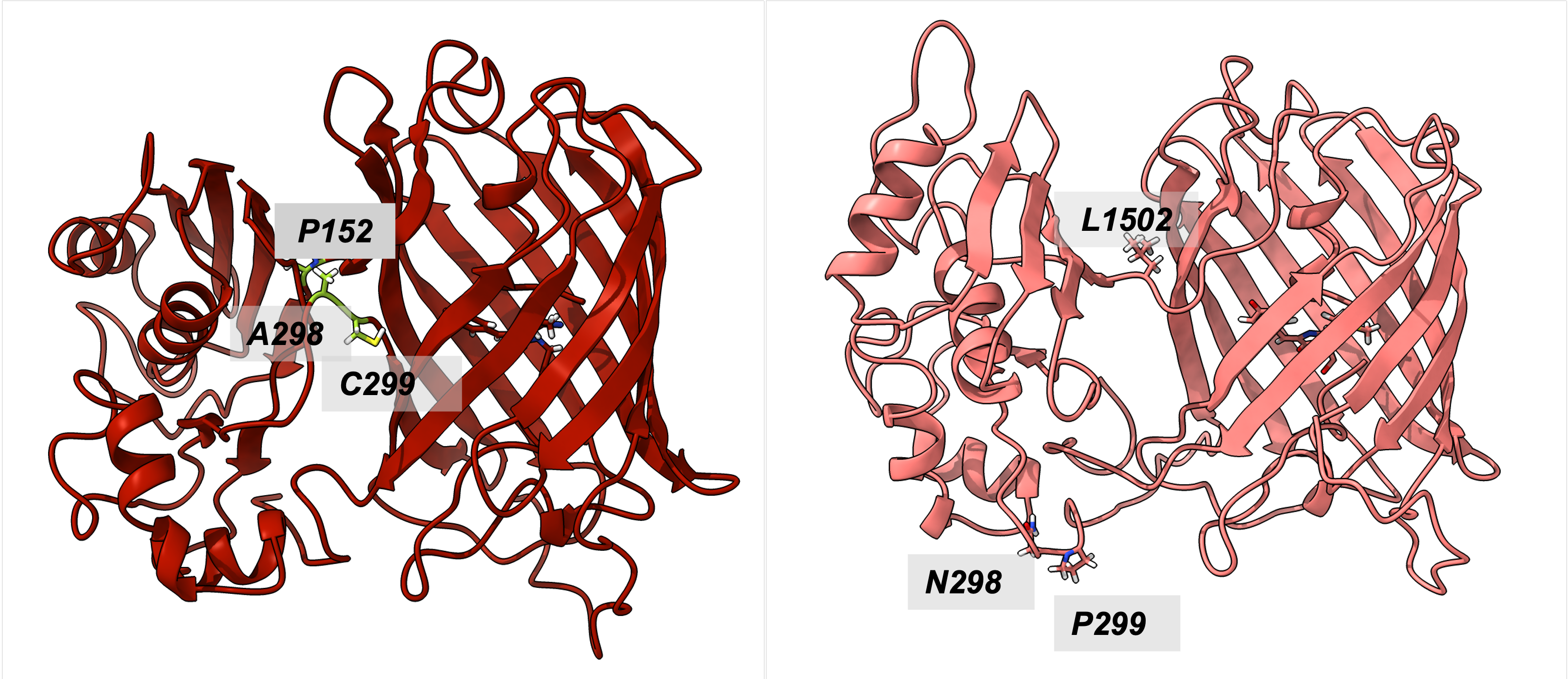

Supplement: S16 Fig — Structure of RGEPO1 (in red) and RGEPO2 (in salmon pink) with mutated positions highlighted in sticks. (TIFF) [file pbio.3002993.s020.tiff]

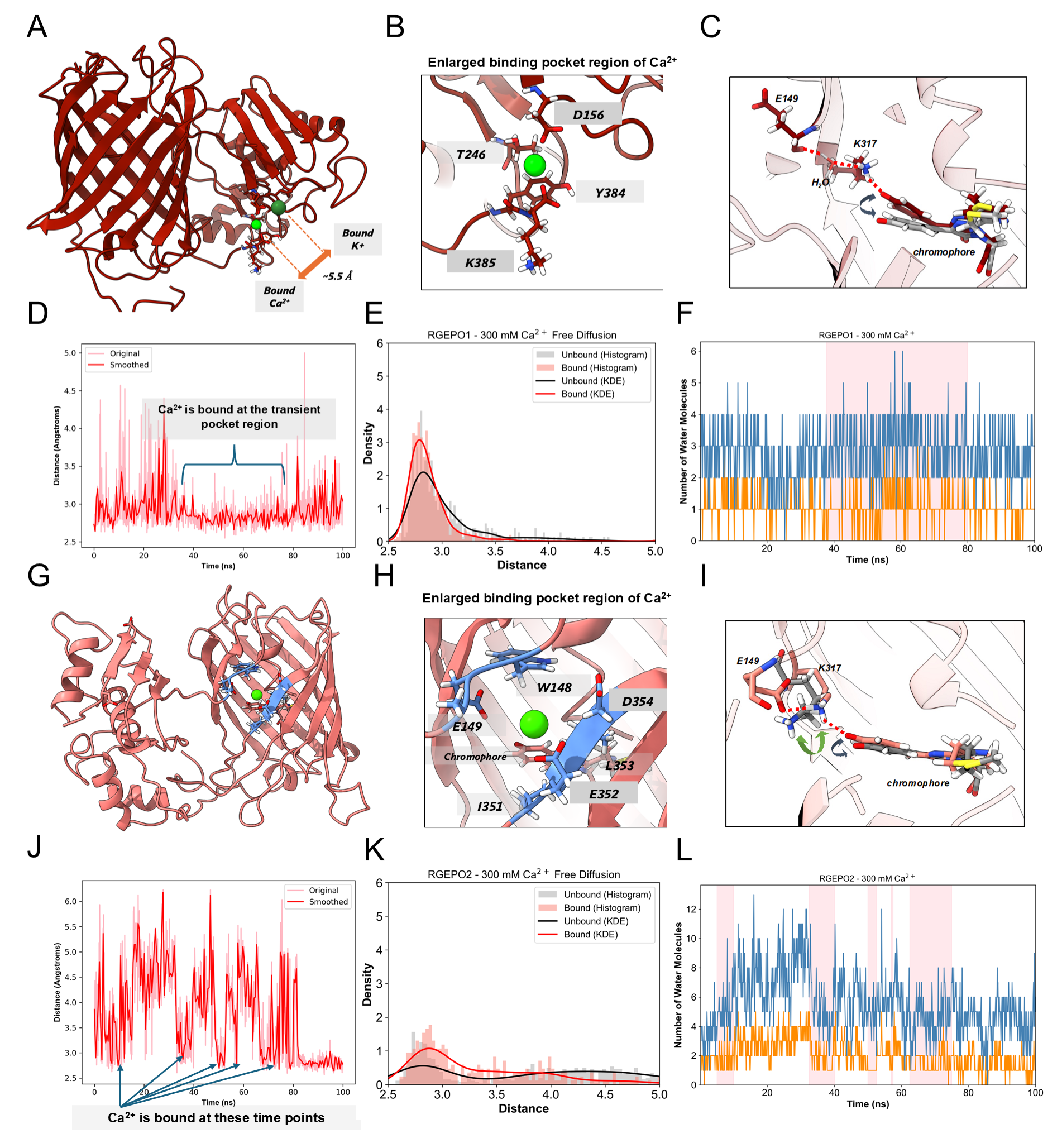

Supplement: S17 Fig — (A) The predicted 3D structure of RGEPO1 is shown in red, with calcium ions represented in light green. Residues surrounding the binding pockets are shown as blue sticks. This binding region is more solvent-exposed compared to the transient pocket observed for K+/Na+ in RGEPO1 (located ~5.5 Å away). (B) Enlarged from (A), showing the main residues at the Ca2⁺ binding pocket. (C) Sensing mechanism of RGEPO1 upon Ca2+ binding. (D) Distance fluctuation between the K317 side chain nitrogen and the phenolate oxygen of the chromophore over time and Ca2⁺-bound intervals are marked with brackets. (E) Density plot generated using the distance data in (D), illustrating bound and unbound Ca2⁺ states. (F) Number of water molecules within 3.5 Å (orange) and 5 Å (blue) from the chromophore’s O-position throughout the simulation. Periods with bound Ca2⁺ are highlighted in pink. (G) The predicted 3D structure of RGEPO2 is shown in pink, with calcium ions represented in light green. Residues surrounding the binding pockets are shown as blue sticks. (H) Enlarged from (G), showing the main residues at the Ca2⁺ binding pocket. (I) Sensing mechanism of RGEPO2 upon Ca2+ binding. (J) Distance variation between K317 side chain nitrogen and chromophore phenolate oxygen with time and Ca2+ bound time period is highlighted with a bracket. (K) Density plot made by using the distance variation data of graph (J), showing the bound and unbound variation. (L) Variation in the number of water molecules surrounding the chromophore O-position within 3.5 Å (orange) and 5 Å (blue) throughout the simulation trajectory. Time periods during which a Ca2⁺ ion is bound are highlighted in pink. (TIFF) [file pbio.3002993.s021.tiff]

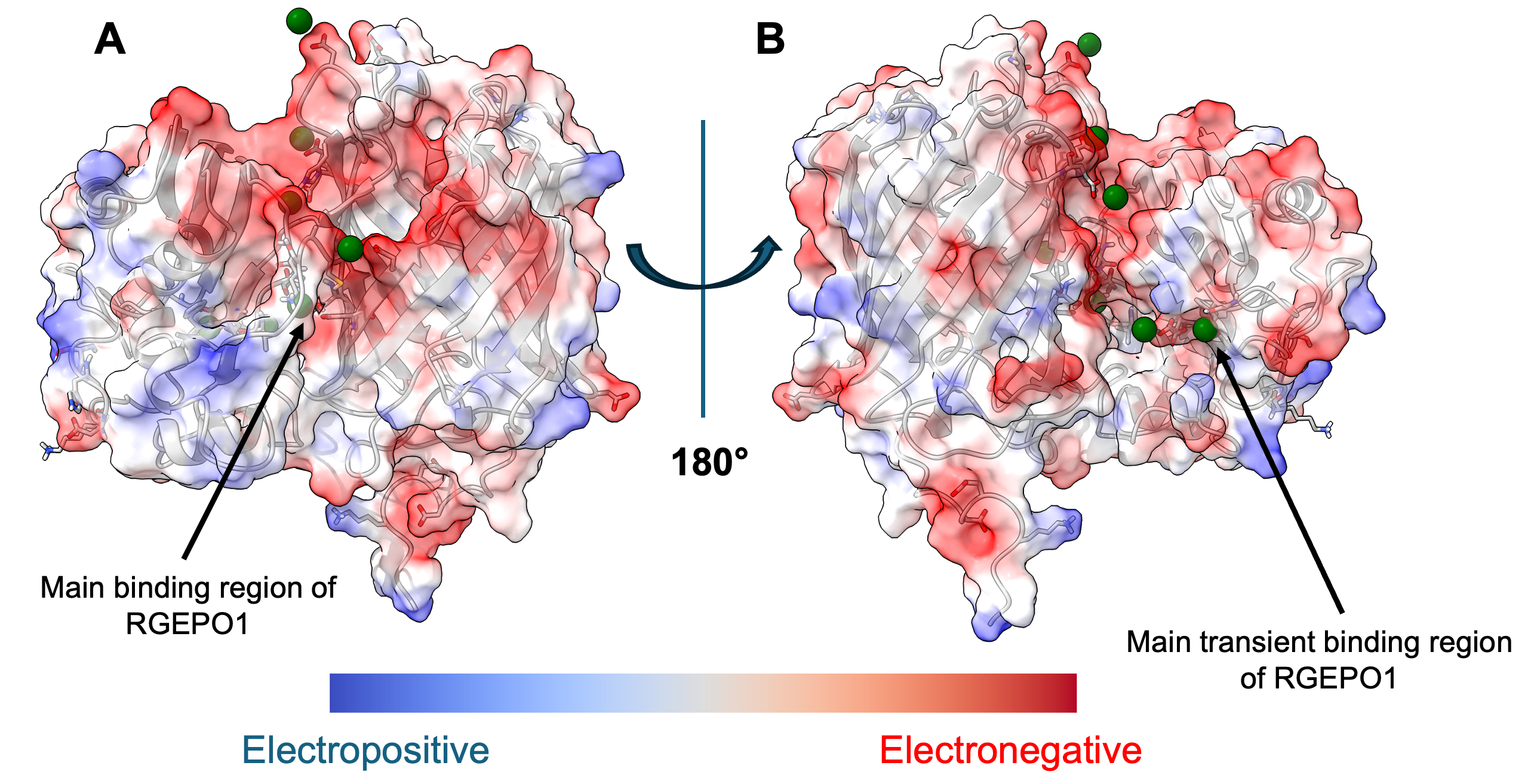

Supplement: S18 Fig — Figures a and b show the electrostatic charge distribution on the surface of RGEPO1 (A: front view, B: back view after a 180° rotation). In these figures, red represents more negatively charged regions, while blue represents more positively charged regions, including both potential transient sites and main binding sites (labeled with an arrow) as observed in free diffusion simulations with K+ shown in green spheres. (TIFF) [file pbio.3002993.s022.tiff]

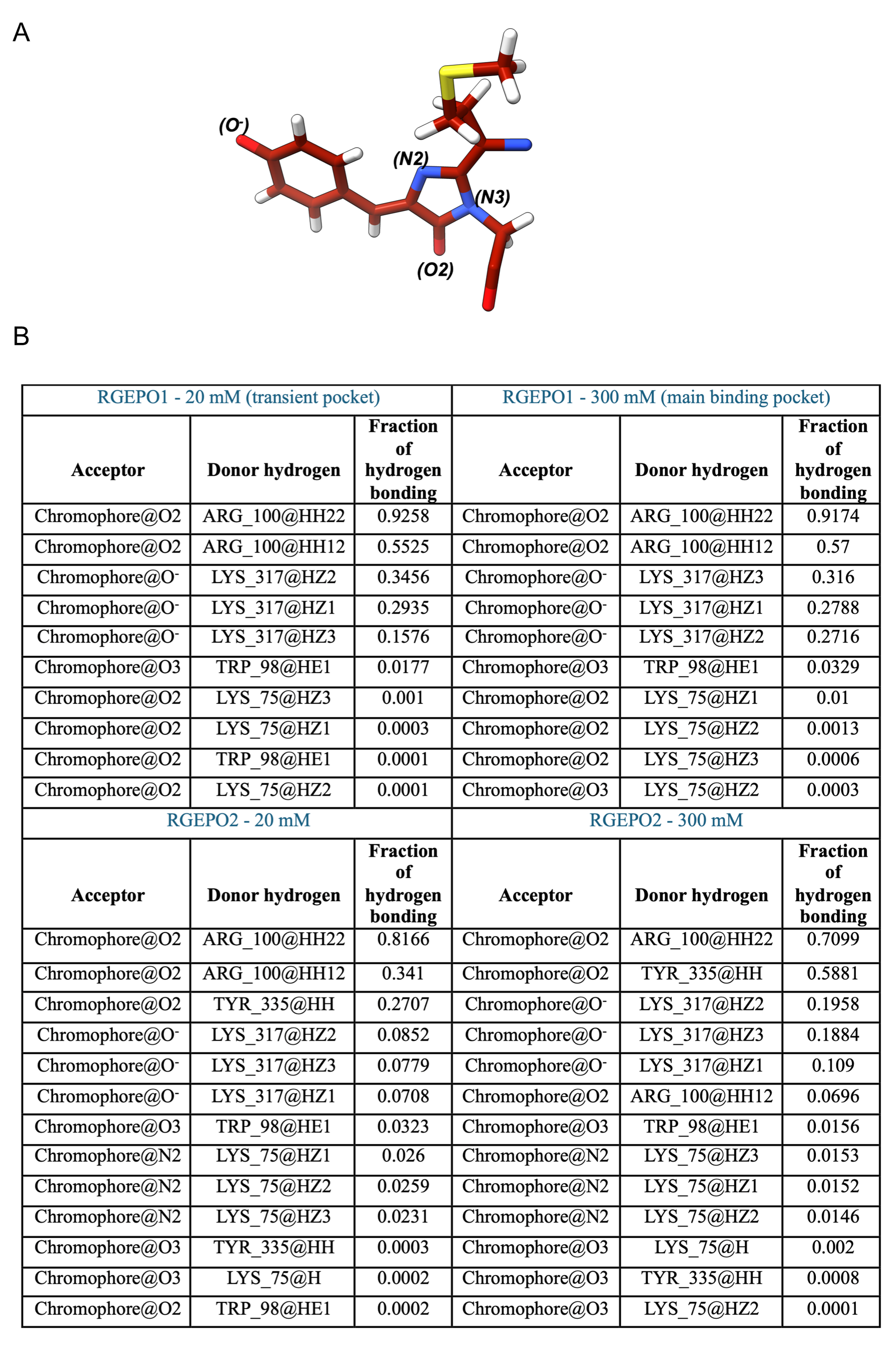

Supplement: S19 Fig — (A) Atom names of the chromophore and (B) table of hydrogen bonding analysis. (TIFF) [file pbio.3002993.s023.tiff]

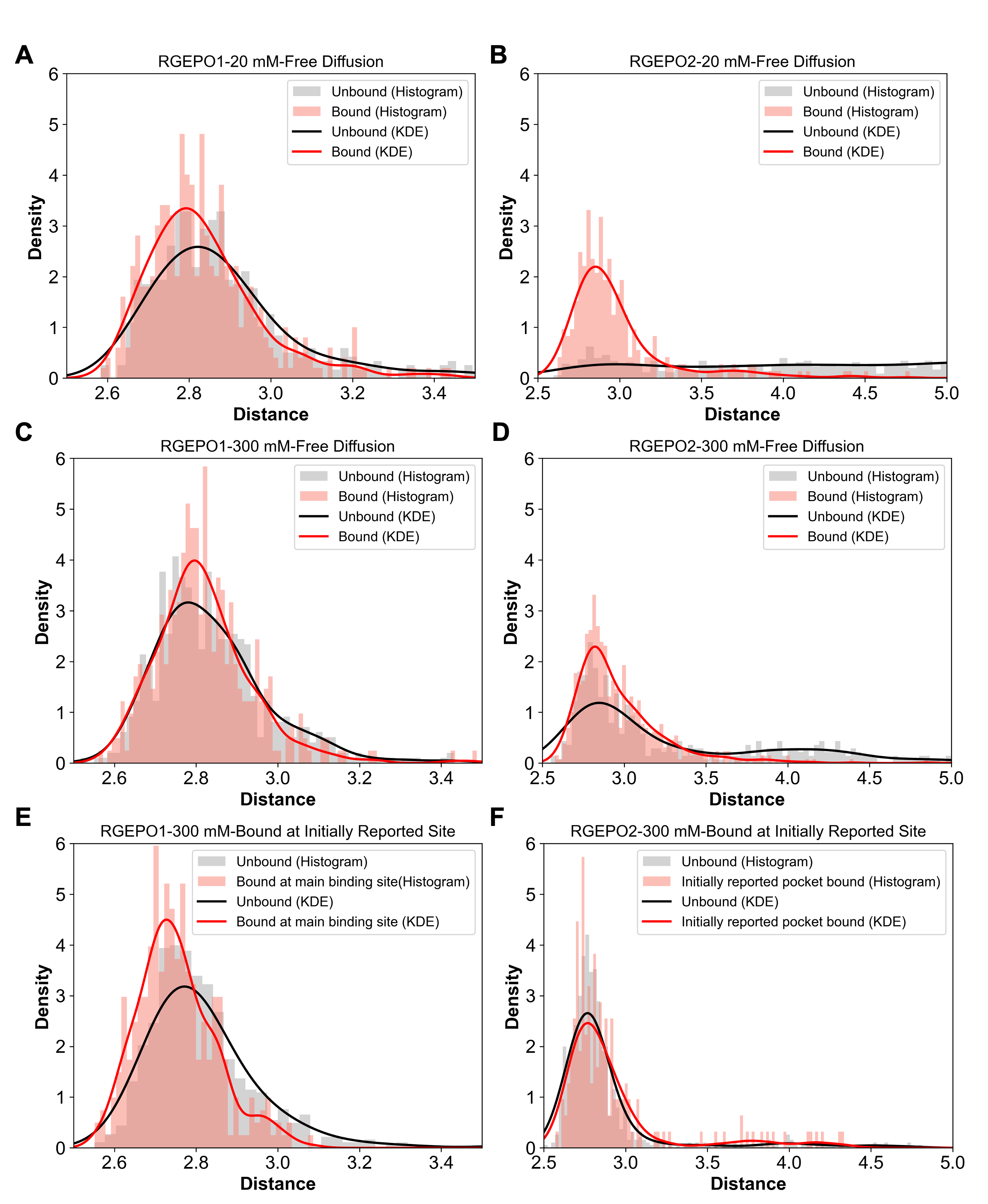

Supplement: S20 Fig — Graph (A) shows the density plot for the variation in distance between the K317 side chain nitrogen and the O− of the chromophore, where free diffusion of K+ occurs at the transient binding site of RGEPO1 at 20 mM concentration. Graphs (B) to (D) display the density plots for the free diffusion simulations of K+ at the main binding sites of RGEPO1 and RGEPO2, as labeled. Graphs (E) and (F) correspond to K+ bound simulations at the initially reported Kbp-K site. In graph (E), the analysis focuses on the free diffusion of K+ at the main binding site, while in graph (F), it pertains to K+ at the initially reported site. The underlying numerical data for this figure can be found in S1 Data. (TIFF) [file pbio.3002993.s024.tiff]

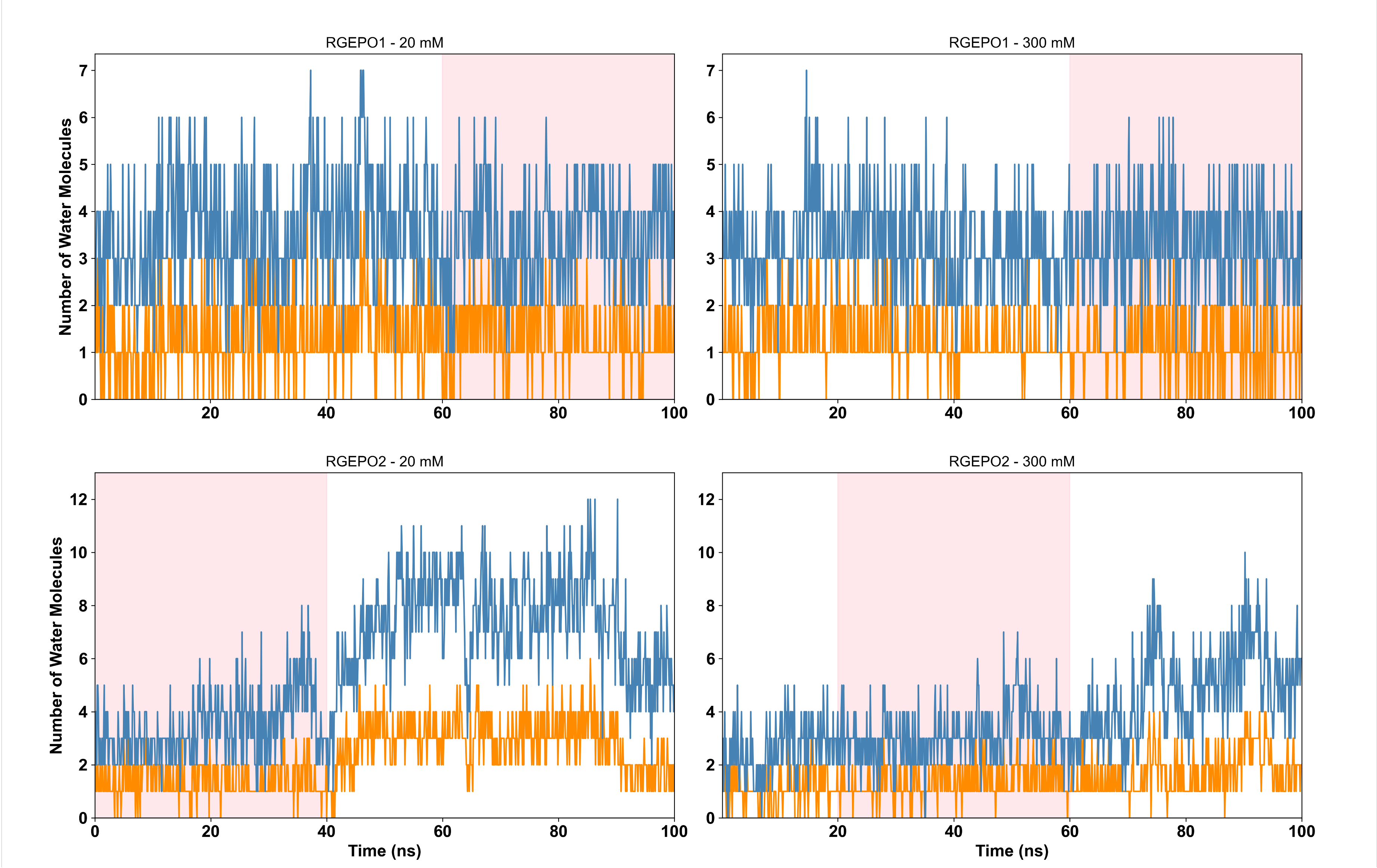

Supplement: S21 Fig — These graphs depict the change in the number of water molecules around the O− position within 3.5 Å (orange) and 5 Å (blue) throughout the trajectory. The time periods during which a K+ ion is bound are highlighted in pink. All simulations are for free diffusion of K+ , with binding occurring at the main binding site. However, the simulation for RGEPO1 at 20 mM concentration specifically focuses on the transient binding of K+. The underlying numerical data for this figure can be found in S1 Data. (TIFF) [file pbio.3002993.s025.tiff]

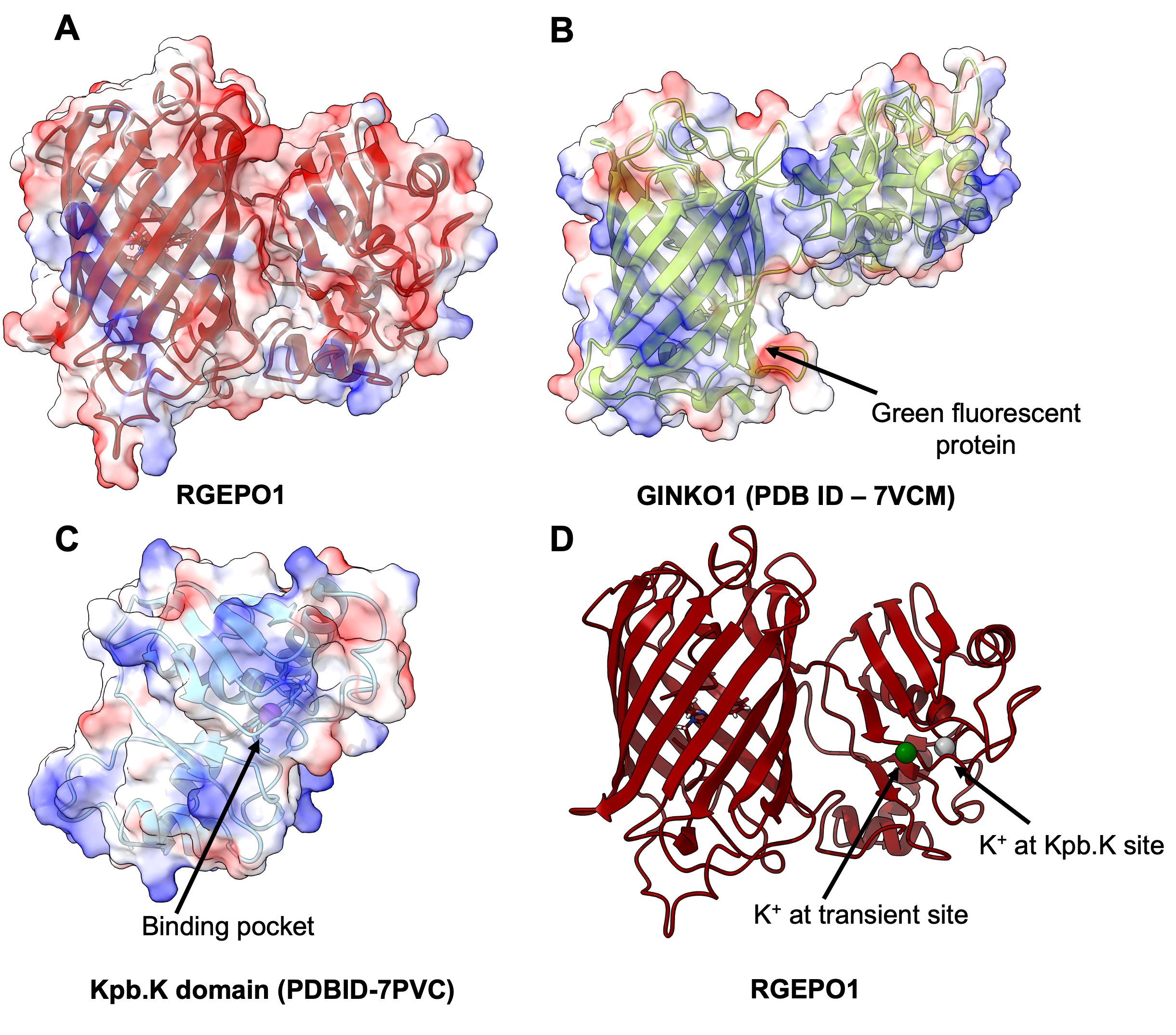

Supplement: S22 Fig — (A) The electrostatic charge distribution of RGEPO1 is shown, with red indicating more electronegative regions and blue representing more electropositive regions. (B) The electrostatic charge distribution of GINKO1 (genetically encoded potassium ion biosensor) (PDB ID – 7VCM) is shown, with red indicating more electronegative regions and blue representing more electropositive regions. (C) The electrostatic charge distribution of the Kbp.K domain (PDB ID: 7PVC) is shown, with red indicating more electronegative regions and blue representing more electropositive regions. The bound K+ is depicted as a purple sphere. (D) The structure of RGEPO1 is shown in red, with the transiently bound K+ in a green sphere and the K+ at the Kbp.K site (PDB ID: 7PVC) in a gray sphere. (TIFF) [file pbio.3002993.s026.tiff]

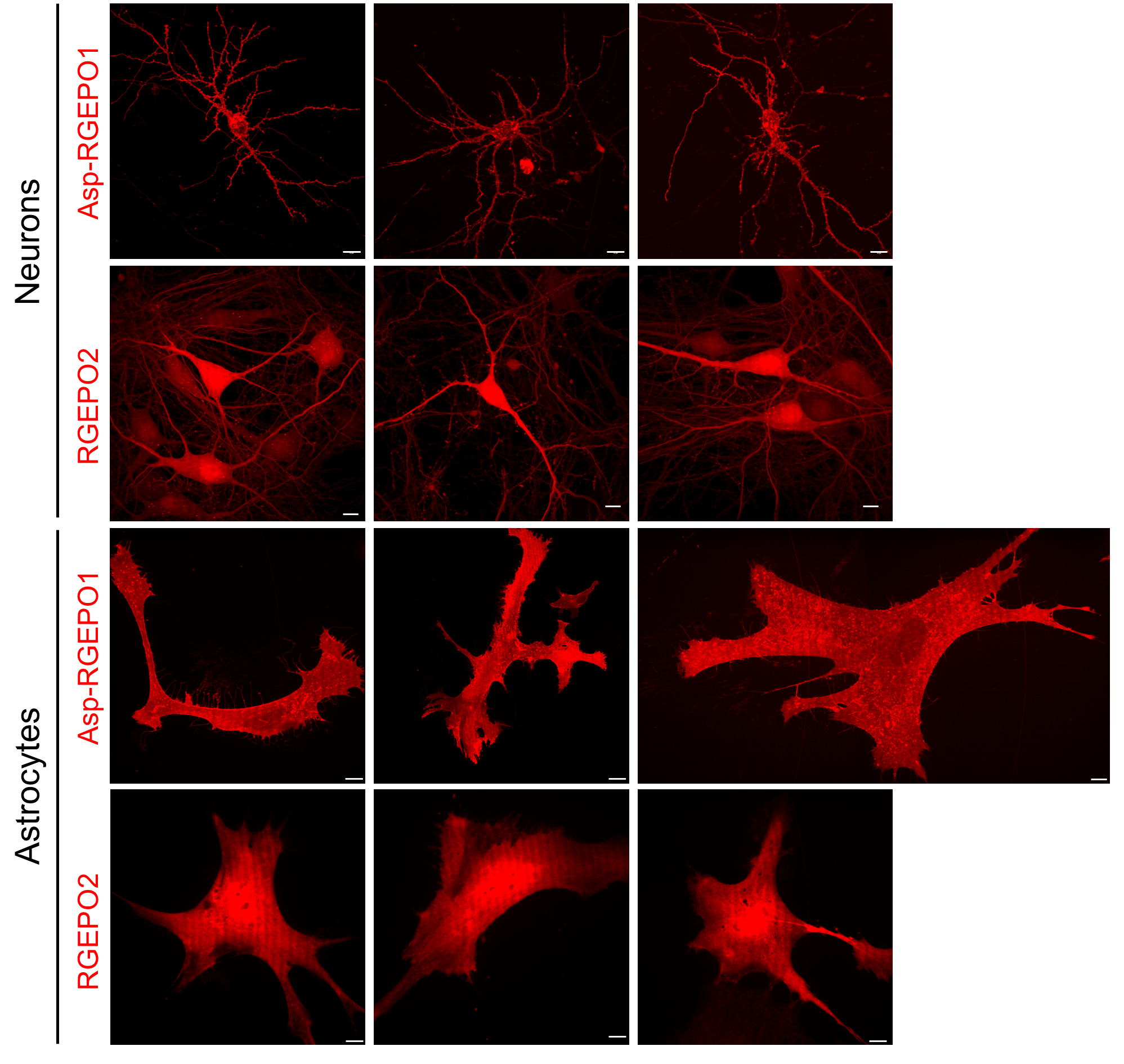

Supplement: S23 Fig — Maximum intensity projection confocal images of Asp-RGEPO1 on the extracellular surface and RGEPO2 in the cytoplasm of live cultured neurons and astrocytes in ACSF buffer (n = 4 and 17 neurons from 2 independent cultures; n = 25 and 17 astrocytes from 2 independent cultures). Scale bars, 10 µm (scale bar of the second image of Asp-RGEPO1on the astrocyte is 30 µm). (TIFF) [file pbio.3002993.s027.tiff]

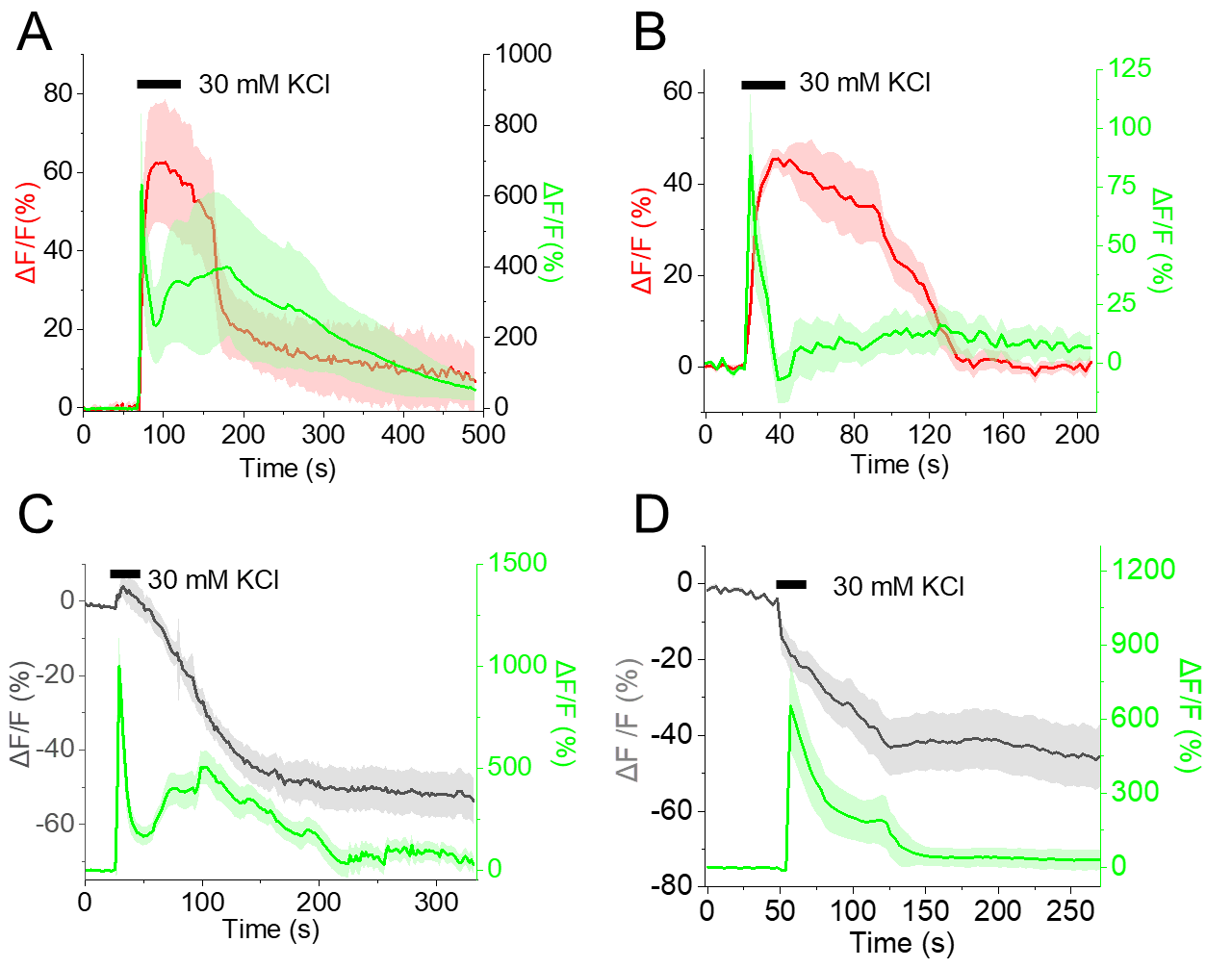

Supplement: S24 Fig — (A, B) Time course of fluorescence intensity changes of Asp-RGEPO1 and GCaMP6f with stimulation of 30 mM KCl on neurons. Each trace represents data from an independent culture (n = 13 and 4 neurons, respectively). Data are shown as mean ± SD. (C, D) Time course of fluorescence intensity changes of RGEPO2 and GCaMP6f with stimulation of 30 mM KCl in neurons. Each trace represents data from an independent culture (n = 38 and 31 neurons, respectively). Data are shown as mean ± SD. The underlying numerical data for this figure can be found in S1 Data. (TIFF) [file pbio.3002993.s028.tiff]

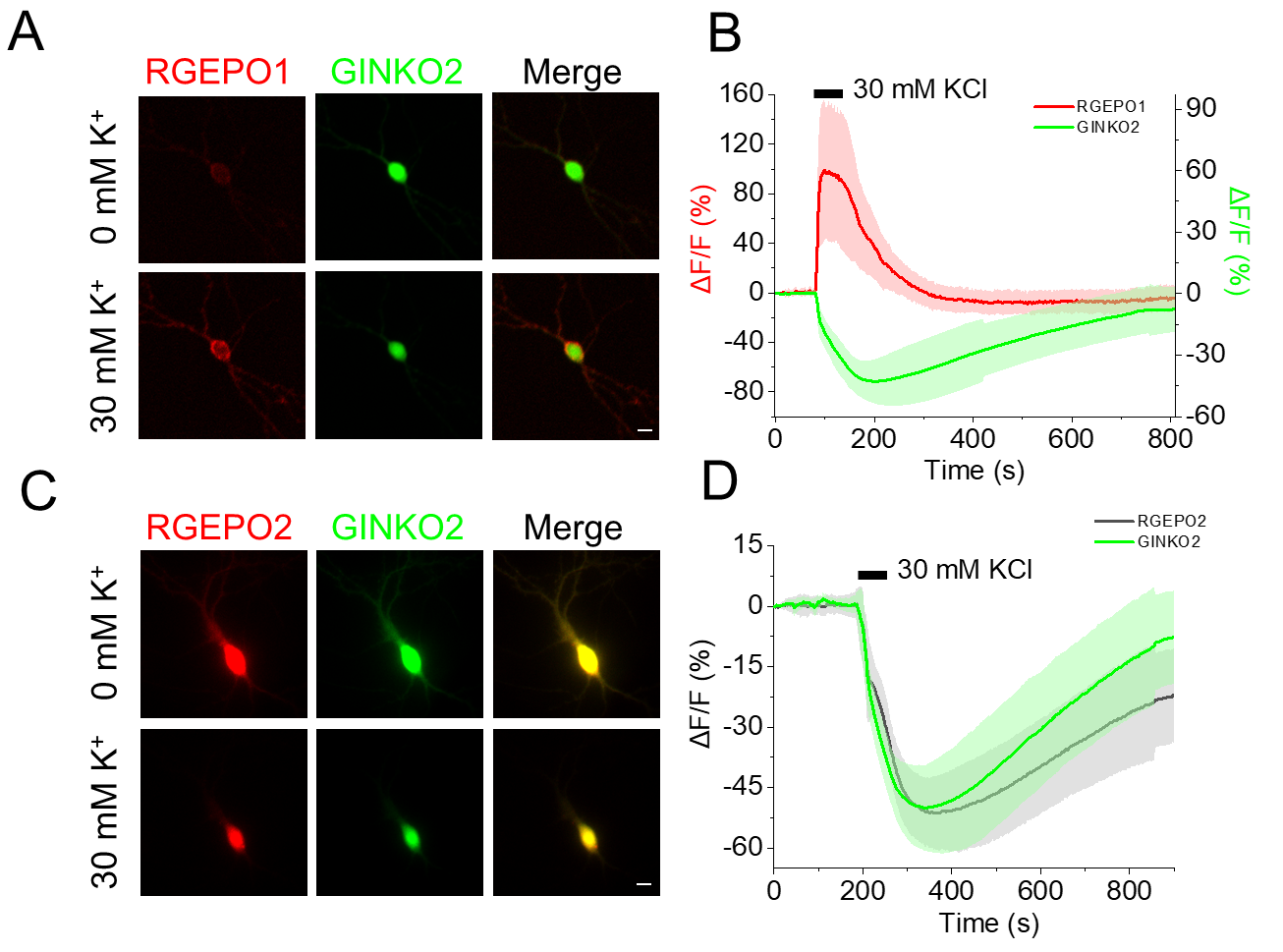

Supplement: S25 Fig — (A) Representative dual-color wide-field imaging of RGEPO1 and GINKO2 dynamics in dissociated hippocampal neurons with response to 30 mM KCl. (B) Time course of fluorescence intensity changes of RGEPO1 and GINKO2 with stimulation of 30 mM KCl in neurons (n = 22 neurons from 3 independent cultures). (C) Representative dual-color wide-field imaging of RGEPO2 and GINKO2 dynamics in dissociated hippocampal neurons with response to 30 mM KCl. (D) Time course of fluorescence intensity changes of RGEPO2 and GINKO2 with stimulation of 30 mM KCl in neurons (n = 51 neurons from 3 independent cultures). The underlying numerical data for this figure can be found in S1 Data. (TIFF) [file pbio.3002993.s029.tiff]

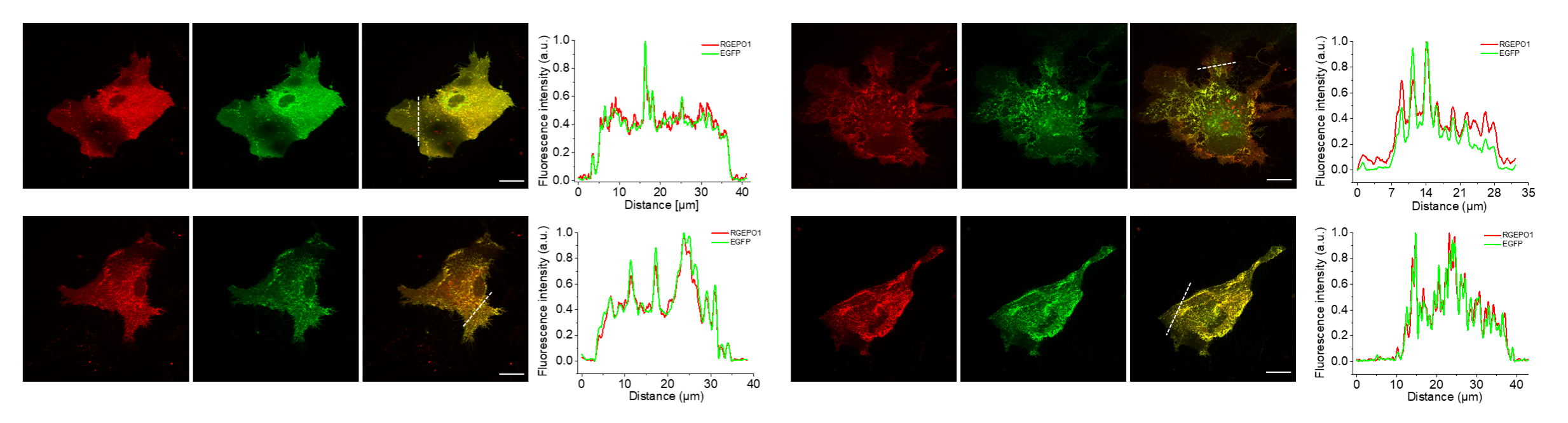

Supplement: S26 Fig — Fluorescence images and membrane localization analysis of Asp-RGEPO1 expressed on the extracellular surface of astrocytes. Membrane-targeted EGFP (Igκ-EGFP) was co-expressed to label the plasma membrane. Left, single-plane confocal fluorescence images of astrocytes expressing the RGEPO1 (red) and EGFP (green) (n = 4 cells from one independent culture). Right, normalized linecut (shown as white dashed line on the left) plots of fluorescence signals measured in both the red and green channels. Scale bars, 20 µm. The underlying numerical data for this figure can be found in S1 Data. (TIFF) [file pbio.3002993.s030.tiff]

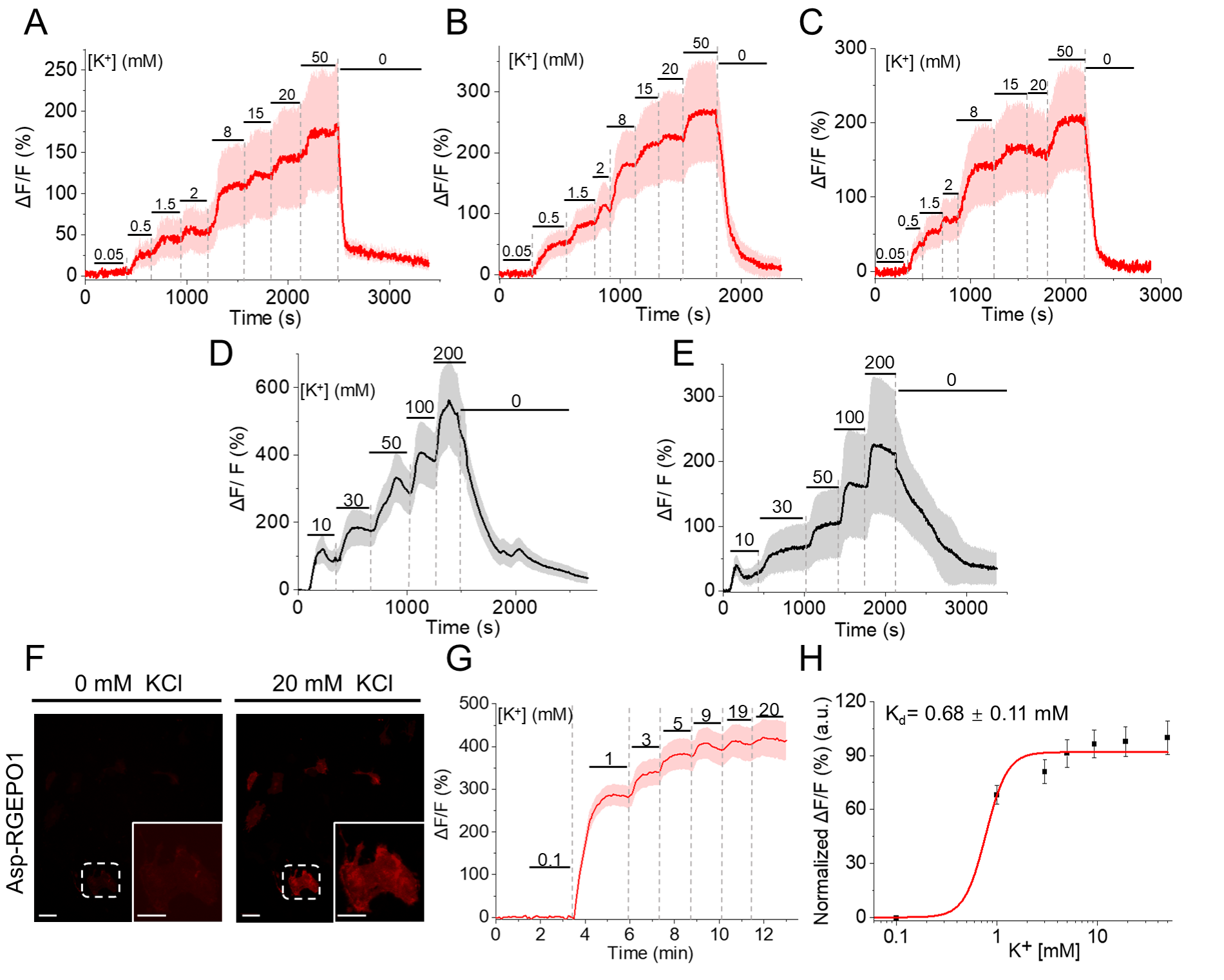

Supplement: S27 Fig — (A–C) Time courses of fluorescence intensity change (ΔF/F) of RGEPO1 on astrocytes stimulated with a series of K⁺ buffers. Each trace represents data from an independent culture (n = 11, 11 and 13 astrocytes, respectively). Data are shown as mean ± SD. (D, E) Time courses of fluorescence intensity change (ΔF/F) of RGEPO2 in astrocytes stimulated with a series of K⁺ buffers. Each trace represents data from an independent culture (n = 8 and 14 astrocytes, respectively). Data are shown as mean ± SD. (F) Representative fluorescence images of primary astrocyte expressing RGEPO1 in response to 20 mM KCl (n = 25 astrocytes from 2 independent cultures). Scale bars, 100 µm, insert, 20 µm. (G) Time course of fluorescence intensity changes of RGEPO1 with stimulation by a series of KCl buffers on astrocytes (n = 6 astrocytes from 1 culture), data are expressed as mean ± SD. The titration was performed by manual addition of each buffer. (H) Plot of normalized ∆F/F against different K+ concentrations fitted using nonlinear fitting (Hill) for the data shown in panel G. The underlying numerical data for this figure can be found in S1 Data. (TIFF) [file pbio.3002993.s031.tiff]

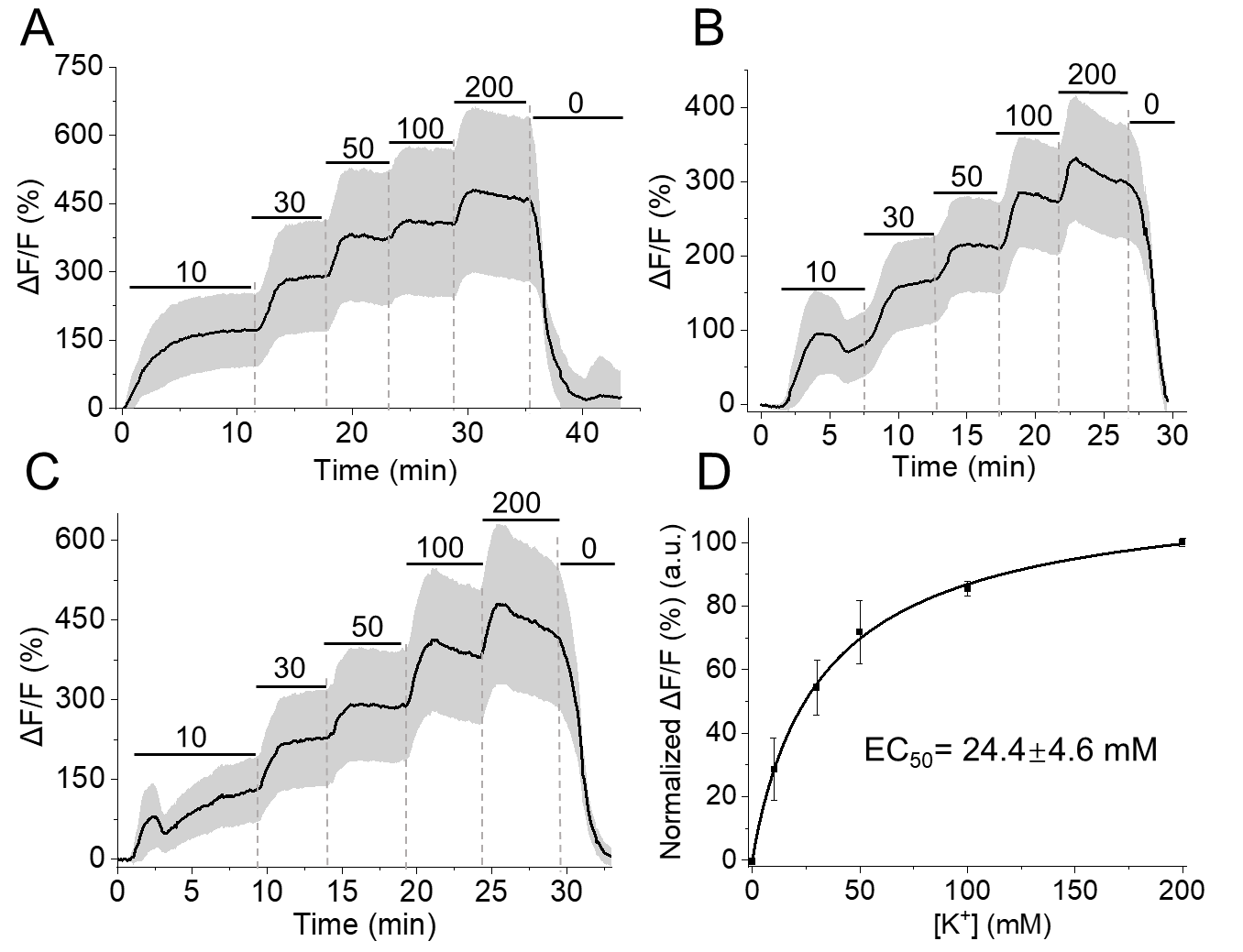

Supplement: S28 Fig — (A, B) Time courses of fluorescence intensity change (ΔF/F) of RGEPO2 in the astrocytes stimulated with a series of K⁺ buffers. Panel (A) represents data from one independent culture (n = 12 cells), and panels B and C are from another independent culture (n = 6 and 5 cells, respectively). Data are shown as mean ± SD. (B) Plot of normalized ∆F/F against different K+ concentrations fitted using nonlinear fitting (Hill) for the data shown in panels A, B, and C. The underlying numerical data for this figure can be found in S1 Data. (TIFF) [file pbio.3002993.s032.tiff]

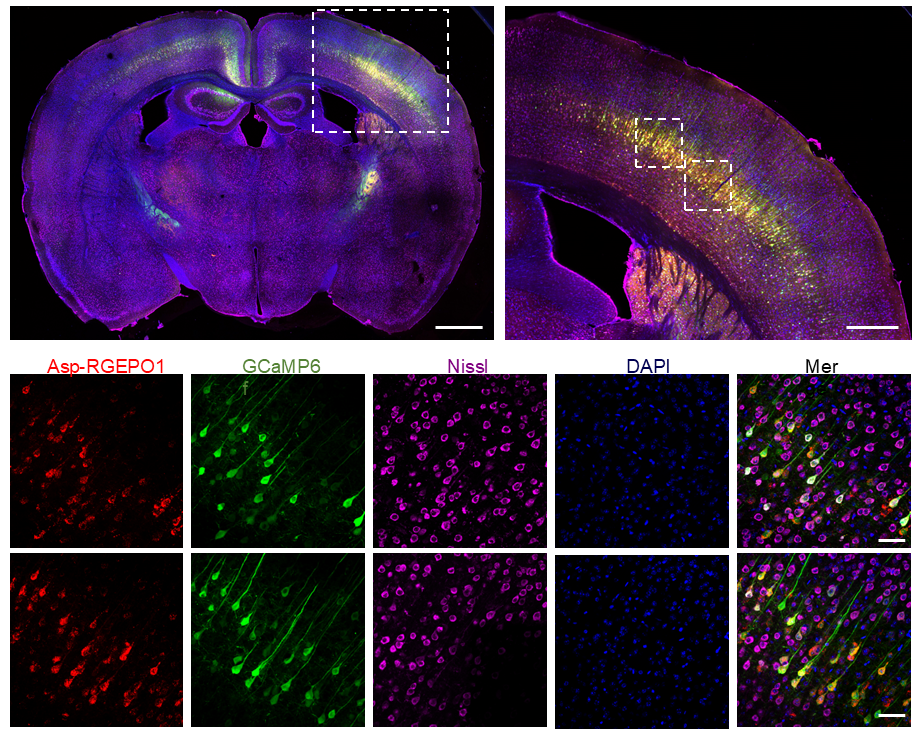

Supplement: S29 Fig — Upper left, single-panel confocal fluorescence image of the cerebral cortex displaying the expression of Asp-RGEPO1 (red) (scale bar = 1,000 µm). Upper right, higher magnification of single panel confocal fluorescence images highlighting relative expression regions (scale bar = 500 µm). Lower, further magnified multi-panel confocal fluorescence images showing detailed expression regions (scale bar = 50 µm). (TIFF) [file pbio.3002993.s033.tiff]

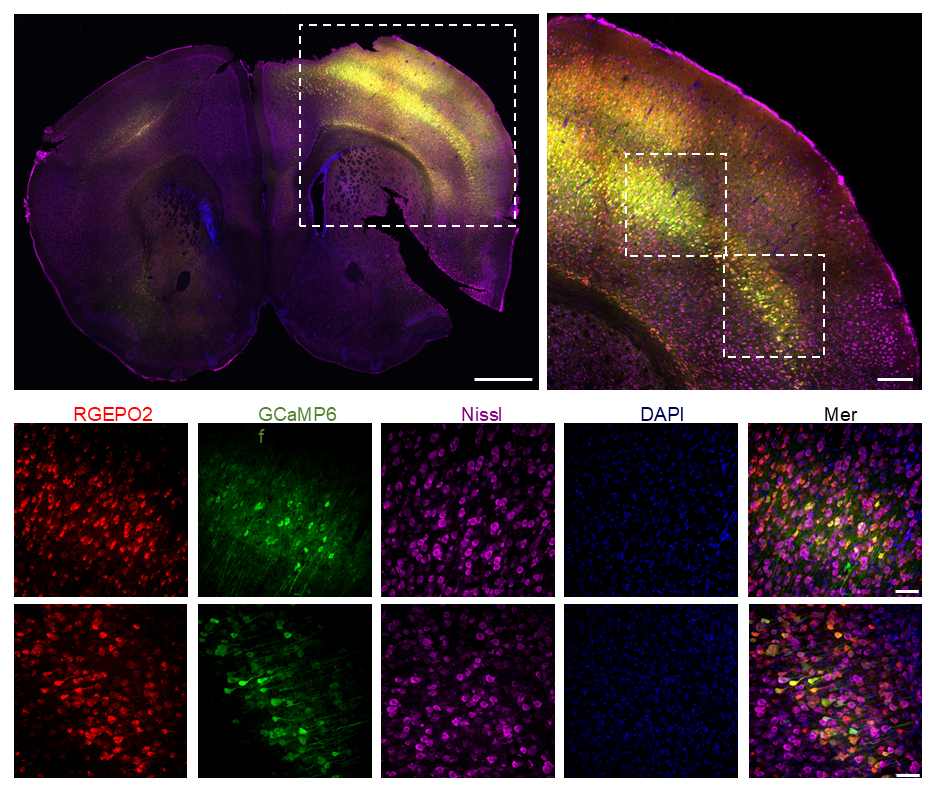

Supplement: S30 Fig — Upper left, single-panel confocal fluorescence image of the cerebral cortex displaying the expression of RGEPO1 (red) and GCAMP6f (green) (scale bar = 1,000 µm). Upper right, higher magnification of single panel confocal fluorescence images highlighting relative expression regions (scale bar = 200 µm). Lower, further magnified multi-panel confocal fluorescence images showing detailed expression regions (scale bar = 50 µm). In this representative image, the expression of RGEPO2 appears stronger in the right hemisphere and weaker in the left. Although bilateral AAV injections were performed, the observed asymmetry in expression intensity may be due to slight differences during manual injection, such as pipette placement, angle, or tissue backflow. Additionally, the selected section may have included more of the expression region in the right hemisphere. These technical differences are commonly encountered in intracerebral viral delivery and do not affect our main conclusions. (TIFF) [file pbio.3002993.s034.tiff]

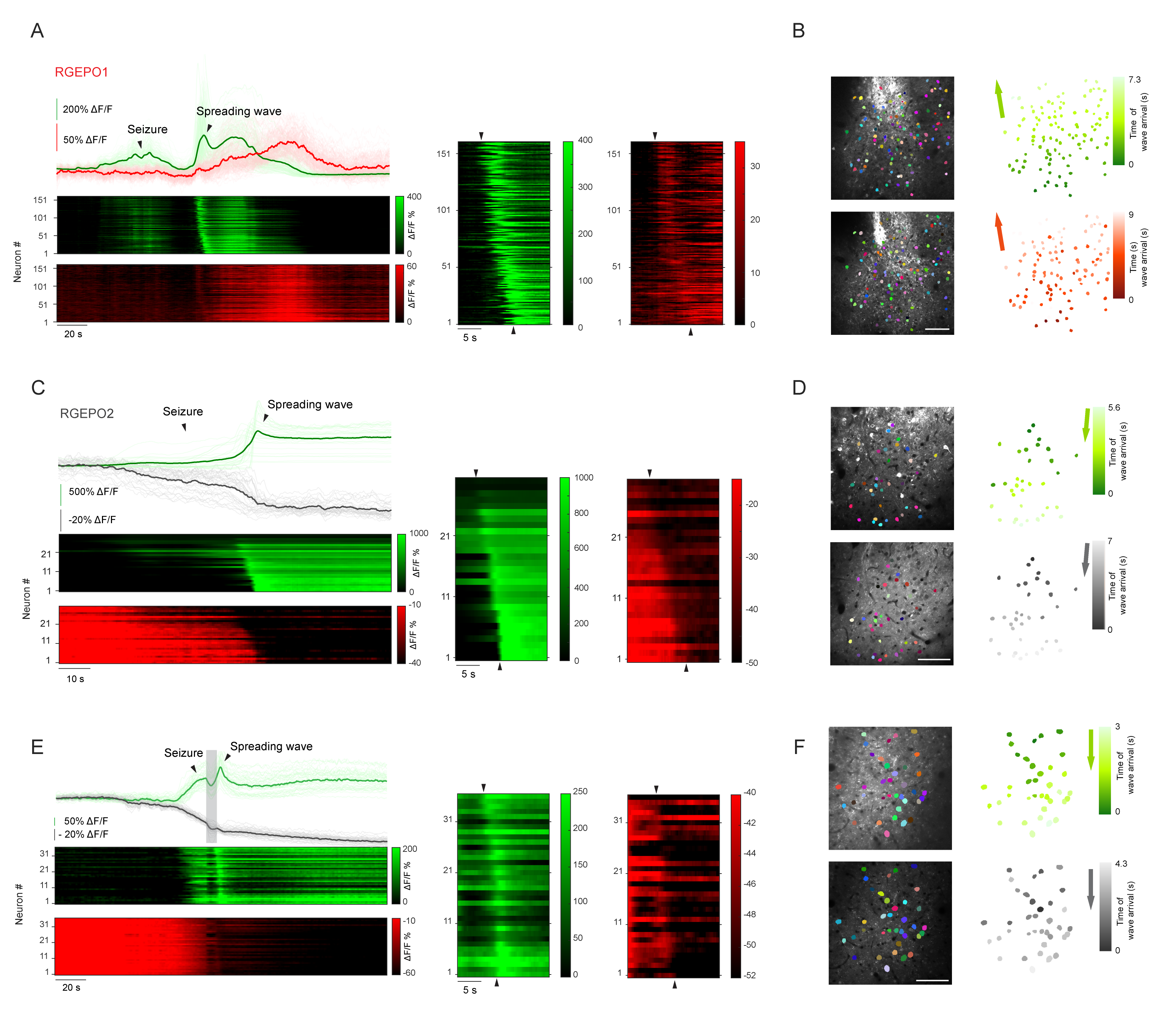

Supplement: S31 Fig — (A) Fluorescence response traces from a mouse co-expressing GCaMP6f and RGEPO1, with vertical projections of neuronal fluorescence profiles during KA-induced seizures and subsequent spreading waves, n = 160 neurons from one mouse. Right panels represent zoomed-in view of the vertical line profile of GCaMP6f and RGEPO2, highlighting the onset of the spreading wave. (B) Temporal analysis of the spreading wave of GCaMP6f and RGEPO1 following seizure activity. Left: pseudo-colored mask of individual neurons overlaid on the average projection image. Right: same mask as left, colored based on fluorescence peak time, n = 111 neurons from one mouse. (C) Fluorescence response traces from a mouse co-expressing GCaMP6f and RGEPO2, with vertical projections of neuronal fluorescence profiles during KA-induced seizures and subsequent spreading waves, n = 160 neurons from one mouse. (D) Temporal analysis of the spreading wave of GCaMP6f and RGEPO2 following seizure activity, n = 29 neurons from one mouse. (E, F) Similar to (C, D), but for another mouse co-expressing GCaMP6f and RGEPO2. n = 36 neurons from one mouse. The gray shaded box indicates the period of acute motion observed along the z-axis. Colored arrow indicates the estimated direction of wave propagation. Scale bar: 100 µm. The underlying numerical data for this figure can be found in S1 Data. (TIF) [file pbio.3002993.s035.tif]
