# Supplementary material for: Sensitive red fluorescent indicators for real-time visualization of potassium ion dynamics in vivo
Source: PLoS Biol. 2025 Sep 17;23(9):e3002993. doi: 10.1371/journal.pbio.3002993 (PMC12456824; doi:10.1371/journal.pbio.3002993)
Supplement: Attachment — Submitted filename: review_PBIOLOGY_20250123.docx [file pbio.3002993.s045.docx]

In this manuscript, Yang et al. present the development of two red genetically encoded potassium indicators (RGEPOs), RGEPO1 and RGEPO2, for studying K^+^ dynamics in biological systems. The authors employed a robust strategy combining directed evolution and mammalian cell optimization to efficiently generate these novel sensors. Comprehensive characterization of RGEPOs was performed, along with mechanistic insights into K^+^ binding through structural analysis. Subsequently, the simultaneous recording of K^+^ and Ca^2+^ signals using RGEPOs and GCaMP6f in diverse settings, including cultured neurons, astrocytes, brain slices, and *in vivo*, demonstrates the significant potential of these tools. The development of RGEPOs provides a valuable toolkit for investigating the roles of potassium in various physiological and pathological processes.

My critiques are as follows:

1. Photoactivation Concerns: Given that RGEPOs are based on mApple, a known concern is potential blue-light-induced photoactivation, which could interfere with the use of blue-light-activated optogenetic tools. Have the authors test whether RGEPOs exhibit such phenomenon? This information is crucial for researchers considering the combined use of these sensors with optogenetics.
2. Refinement of Characterization Data: Certain characterization results could benefit from further optimization for enhanced precision. In Fig 2h, extending the measurement duration under each K^+^ concentration would ensure the fluorescence intensity reaches a stable level, providing more robust data. In Fig 2i & 5i, a denser sampling of K^+^ concentrations around the calculated Kd would improve the accuracy of the dose-response curve. Additionally, the fit of RGEPO2 curve in Fig 3f appears suboptimal, suggesting a need for careful evaluation and potential refinement of the EC50 value.
3. Two-Photon Excitation Spectrum: Since two-photon microscopy was utilized (Fig 7), including the two-photon excitation spectrum of RGEPOs in a supplementary figure would be highly beneficial for researchers selecting appropriate laser wavelengths for their experiments.
4. Standardization of Data Presentation: The use of log scale in Fig 2i & 5i while employing linear scale in Fig 2l & 5l may cause confusion. Standardizing the data presentation across these figures would enhance clarity and readability.
