# Supplementary material for: Sensitive red fluorescent indicators for real-time visualization of potassium ion dynamics in vivo
Source: PLoS Biol. 2025 Sep 17;23(9):e3002993. doi: 10.1371/journal.pbio.3002993 (PMC12456824; doi:10.1371/journal.pbio.3002993)
Supplement: Attachment — Submitted filename: Responses to Reviewers 2025-06-22.docx [file pbio.3002993.s047.docx]

Dear Richard,

We are very grateful for the fast and efficient review process of our manuscript. We find all Reviewers’ comments extremely constructive and agree with all of them. In response to the reviewer’s comments, we conducted several new experiments to strengthen the overall characterization and validation of RGEPOs. We first evaluated their photoactivation properties and measured their two-photon excitation spectra to further characterize their photophysical properties. To improve the accuracy of response quantification, we re-titrated RGEPO1 on the extracellular surface of HEK293FT cells and cultured mouse astrocytes, extending the titration range around the EC₅₀ and increasing the recording duration to obtain better curve fitting. We also benchmarked RGEPOs against GINKO2 in cultured primary neurons, confirming their compatibility for dual-color imaging and simultaneous monitoring of potassium dynamics. To refine intracellular K⁺ sensing, we performed gramicidin-based titration in HEK293FT cells, allowing intracellular K⁺ depletion prior to stimulation. In addition, we validated RGEPO2’s responsiveness to physiological activity by co-expressing it with GCaMP6f and applying glutamate in primary mouse neurons. To assess K⁺ selectivity, we conducted Ca²⁺ titration in solution and performed MD simulations, both of which indicated minimal Ca²⁺ interference. We further demonstrated membrane localization of RGEPO1 in astrocytes using co-expression with Igk-EGFP, and included additional biological replicates to enhance statistical robustness. Together, these efforts comprehensively address the reviewer’s concerns and support the robustness and applicability of RGEPOs across various cellular contexts.

We have added 9 new Supplementary Figures and 2 Supplementary Tables, and also updated several original figures with new data. Specifically, in the main text figures, panels in Figure 2g–l were revised to incorporate new results. In Figure 3f, we refined the curve fitting to improve accuracy. Panels in Figure 5b–d, g–i, and l were also updated based on newly acquired data. In Figure 6f, we corrected the label of RGEPO1, and in Figure 7, the panels were revised to reflect updated experimental findings. In the supplementary section, we revised Supplementary Figures 10, 24, and 28 to include more representative traces. Please find below the detailed point-by-point responses to the comments.

**Reviewer #1**:

*In this manuscript, Yang et al. present the development of two red genetically encoded potassium indicators (RGEPOs), RGEPO1 and RGEPO2, for studying K+ dynamics in biological systems. The authors employed a robust strategy combining directed evolution and mammalian cell optimization to efficiently generate these novel sensors. Comprehensive characterization of RGEPOs was performed, along with mechanistic insights into K+ binding through structural analysis. Subsequently, the simultaneous recording of K+ and Ca2+ signals using RGEPOs and GCaMP6f in diverse settings, including cultured neurons, astrocytes, brain slices, and in vivo, demonstrates the significant potential of these tools. The development of RGEPOs provides a valuable toolkit for investigating the roles of potassium in various physiological and pathological processes.*

We sincerely thank Reviewer for their thorough and insightful evaluation of our manuscript. We are grateful for the positive summary of our work and for recognizing the potential value of RGEPOs in studying potassium dynamics in diverse biological contexts. We also appreciate the constructive suggestions, which have helped us further improve the clarity and completeness of the revised manuscript. Below, we provide point-by-point responses to each of the raised concerns and comments.

*My critiques are as follows:*

*1. Photoactivation Concerns: Given that RGEPOs are based on mApple, a known concern is potential blue-light-induced photoactivation, which could interfere with the use of blue-light-activated optogenetic tools. Have the authors test whether RGEPOs exhibit such phenomenon? This information is crucial for researchers considering the combined use of these sensors with optogenetics.*

We thank Reviewer for bringing this up. Indeed, almost all Apple-derived biosensors exhibit considerable photoactivation in a red channel under blue illumination (10.3389/fnmol.2013.00002; 10.1021/cn400012b; 10.1016/j.neuron.2014.11.005). Correspondingly, we tested RGEPOs photoactivation following the standard protocol used for red calcium sensors characterization reported previously by Hod Dana et al (10.7554/eLife.12727). Specifically, photoactivation of RGEPOs was assessed in live HEK293FT cells by continuous imaging under green-light excitation (555/20 nm) combined with five additional blue-light stimulations (470/28 nm, 2.1 mW/mm², 5 s pulse). As a result, we did observe that RGEPOs exhibit a certain degree of blue-light-induced photoactivation (see Figure R1), similar to that reported for jRGECO1a previously.

**Figure R1.** Photoactivation of RGEPO1 (n = 10 cells from one culture) and RGEPO2 (n = 8 cells from one culture) was assessed in HEK293FT cells by continuous imaging with green-light excitation (555/20 nm), combined with five additional blue-light stimulations (470/28 nm, 2.1 mW/mm^2^, 5 s per pulse, as indicated).

We have revised the manuscript to describe new results and corresponding methods, as well as discussed this limitation of the RGEPO sensors in the Discussion section. Please see new Supplementary Figure 11 and lines 201-209 and 403-406 in the result section, lines 618-620 in the discussion section, and lines 811-813 and 847-850 in the method sections of the revised manuscript.

*2. Refinement of Characterization Data: Certain characterization results could benefit from further optimization for enhanced precision. In Fig 2h, extending the measurement duration under each K+ concentration would ensure the fluorescence intensity reaches a stable level, providing more robust data. In Fig 2i & 5i, a denser sampling of K+ concentrations around the calculated Kd would improve the accuracy of the dose-response curve. Additionally, the fit of RGEPO2 curve in Fig 3f appears suboptimal, suggesting a need for careful evaluation and potential refinement of the EC50 value.*

We agree with Reviewer that the characterization results in the original manuscript could be further refined to improve precision. Accordingly, we conducted additional experiments to enhance reproducibility (the revised manuscript now includes results from at least three independent cultures) and added more [K^+^]’s around the estimated EC₅₀, as well as extended the duration of perfusion for each concentration.

To improve consistency and timing control, we replaced manual pipetting used in the original experiments with an automated perfusion system. In this new round of titrations, we extended the duration of prefusion at each K⁺ concentration to allow the fluorescence signal to reach a steady plateau, thereby ensuring the response reflected the stabilized potassium level. The plateau phase at each step lasted approximately two minutes, as shown in the time-course traces in the newly added Figure 2h, Figure 5h, Supplementary Figure 9a-b, and Supplementary Figure 27 a-c.

For Figure 2h, RGEPO1 was expressed on the extracellular surface of HEK293FT cells and titrated using the perfusion system. Intermediate K⁺ concentrations (1.5 mM and 2 mM) were added near the previously estimated EC₅₀, and the EC₅₀ value shifted from 3.55 mM (reported in the original manuscript) to 2.4 mM (now reported in the revised manuscript), which was still within the variability range.

For Figure 5i, we expressed RGEPO1 on the extracellular surface of astrocytes and performed a similar titration with finer concentration steps and extended perfusion durations. The resulting EC₅₀ shifted from 0.68 mM to 4.4 mM. This larger discrepancy is likely attributable to the technical differences in potassium delivery. Manual pipetting, commonly used during early screening, can introduce variability in timing and local concentration, especially under static conditions without active flow, potentially leading to transient local K⁺ enrichment and underestimation of the true EC₅₀.

In contrast, the automated perfusion system ensures consistent buffer exchange, minimizing artifacts caused by variable local concentrations and enabling more accurate and reproducible affinity measurements. This is further supported by the EC₅₀ shift observed in HEK293FT cells under the same methodological change (from 3.55 mM to 2.4 mM), suggesting a general improvement in measurement accuracy.

The relatively larger EC₅₀ shift seen in astrocytes may also reflect cell-type-specific factors, such as greater cellular heterogeneity or differences in membrane accessibility. Thus, while manual pipetting remains suitable for high-throughput screening during the optimization phase, perfusion-based titration provides superior control and reproducibility for final sensor characterization.

For Figure 3f, we also refined the curve fitting using the DoseResp equation instead of the Hill equation used in the original manuscript. While the EC₅₀ value remained practically unchanged, the updated fitting improved the overall curve quality and visual representation in the revised figure.

Please see the revised Figures 2, 3, and 5. Correspondingly, the methods section was revised to describe the changes in the manuscript (please see 182-187 and 450-454 in the result section).

*3. Two-Photon Excitation Spectrum: Since two-photon microscopy was utilized (Fig 7), including the two-photon excitation spectrum of RGEPOs in a supplementary figure would be highly beneficial for researchers selecting appropriate laser wavelengths for their experiments.*

This is a great point. Now, in the revised manuscript, we provided a thorough characterization of the two-photon properties of both RGEPOs in two states, with and without potassium, measured in the protein solution (see Figure R2 below).

**Figure R2.** (a) The 1P spectra of RGEPO1 were recorded under both K⁺-free and K⁺-saturated conditions, represented in blue and red, respectively. Excitation spectra of RGEPO1 are shown as solid lines, and emission spectra as dashed lines. (c) The K⁺-dependent changes of RGEPO1 in 1P fluorescence intensity are shown in black, represented as the ratio of fluorescence under saturated versus free conditions. (b) The 2P excitation spectra of RGEPO1 under K⁺-free (blue) and K⁺-saturated (red) conditions. (d) The K⁺-dependent changes of RGEPO2 in 2P fluorescence intensity are shown in black, represented as the ratio of fluorescence under saturated versus free conditions. (e) The 1P spectra of RGEPO2 were recorded under both K⁺-free and K⁺-saturated conditions, represented in blue and red, respectively. Excitation spectra of RGEPO1 are shown as solid lines, and emission spectra as dashed lines. (g) The K⁺-dependent changes of RGEPO2 in 1P fluorescence intensity are shown in black, represented as the ratio of fluorescence under saturated versus free conditions. (f)The 2P excitation spectra of RGEPO2 under K⁺-free (blue) and K⁺-saturated (red) conditions (h) The K⁺-dependent changes of RGPO2Cin 2P fluorescence intensity are shown in black, represented as the ratio of fluorescence under saturated versus free conditions.

These data will help end users in selecting appropriate laser wavelengths for in vivo experiments. Please see the new Supplementary Figure 13 and the newly added Supplementary Tables 2 and 3. We also described new results in the revised manuscript and added a methods section describing 2P measurements, see lines 240-244 in the results section and lines 746-782 in the methods section.

*4. Standardization of Data Presentation: The use of log scale* *in Fig 2i & 5i while employing linear scale in Fig 2l & 5l may cause confusion. Standardizing the data presentation across these figures would enhance clarity and readability.*

We agree with the Reviewer’s suggestion to unify data representation throughout all figures. Correspondingly, we replotted the log scale in Fig. 2i and Fig. 5i into a linear scale to make it consistent with all other panels. Please see revised Fig. 2 and Fig. 5.

**Reviewer #2**:

*In this study, Yang et al. developed two novel genetically encoded potassium (K+) indicators (GEPOs), which are based on a single (red) fluorescent protein (FP) and on a bacterial Kpb K+ binding domain (Hv-Kbp).*

*GEPOs were engineered to monitor the K+ dynamics in cells and tissues in real-time with the aim of generating tools with a higher dynamic range and stronger signals for multiplex imaging. These newly designed sensors would thus extend our existing tool box of K+ indicators including GEPII and KIRIN, which are based on Förster resonance energy transfer (FRET) as well as GINKO (and several other) that employ single fluorophores of different colors.*

*Based on this background the authors develop a membrane-targeted redGEPO (RGEPO1) designed to detect K+ changes in the extracellular space, while the intracellular expressed redGEPO (RGEPO2) allows examination of K+ dynamics in the cytosol and nucleus.*

*The authors characterize RGEPO1 and RGEPO2 in solution and record their individual fluorescence spectra and fluorescence intensities in the absence (0 mM) and presence of high (150-300 mM) K+ concentrations. Further, they provide evidence for the pH stability and ion selectivity of the engineered K+ biosensors and perform molecular simulations to predict the binding pockets for K+ ions as well as the dynamic changes in charge distributions due to K+ on/off. Upon expression of the RGEPOs in hippocampal neurons and astrocyte cultures in vitro the responsiveness of the biosensors to KCl (in a defined concentration range) was tested to establish their functionality in living mammalian cells. Also, K+ dynamics were studied in acute brain slices and in parallel to Ca2+ changes recorded by GCaMP6f. Finally, RGEPOs and GCaMP6f were employed during kainic acid (KA)-induced seizures in mice providing evidence that these new class of K+ biosensors may allow examination of disease-relevant changes in ion dynamics during epileptiform activity of neurons.*

We sincerely thank Reviewer for the thoughtful and constructive evaluation of our manuscript. We greatly appreciate Reviewer’s clear summary of our study and the recognition of its potential contribution to the field of genetically encoded potassium indicators. We also value the detailed feedback, which has helped us improve the clarity, precision, and overall quality of the revised manuscript. Below, we provide point-by-point responses to each of the comments, outlining the changes we have made in the revised manuscript.

*The data regarding the generation, optimization and application of RGEPO1 and RGEPO2 is - for the most part - presented in a comprehensible manner and the experimental approaches and conclusions are convincing. Yet, I have some important points that refer to the validation of the sensors in the mammalian cells systems utilized that should be addressed prior to publication:*

We are grateful to Reviewer for the constructive comments. To address the raised concerns, we conducted several additional experiments and data analyses. Specifically, we benchmarked RGEPOs against GINKO2 by co-expressing them in cultured neurons, confirming their compatibility for dual-color imaging and simultaneous monitoring of potassium dynamics. We titrated RGEPO2 in HEK293FT cells using gramicidin, which efficiently lowered intracellular K⁺. In this experiment, we observed a stronger ΔF/F response and a higher EC₅₀, more consistent with solution-based measurements than those obtained using valinomycin and CCCP. We further validated RGEPO2's response to the glutamate stimulation by co-expressing it with GCaMP6f in cultured neurons. To further assess ion selectivity, we performed Ca²⁺ titration in solution and MD simulations, both indicating minimal Ca²⁺ interference. We also included additional biological replicates to improve statistical reliability. Finally, we confirmed membrane localization of RGEPO1 in astrocytes through co-expression with Igk-EGFP. Collectively, these additions strengthen the conclusions and demonstrate the robustness and applicability of RGEPOs in diverse settings.

*Major*

*1)      The newly developed RGEPO1 and 2 sensors should be compared side-by-side to existing K+ biosensors such as GEPII or GINKO in a relevant mammalian cell setting e.g. primary neurons. This will allow the reviewer to assess whether two main objectives of the study (besides multiplex analyses) were achieved as the authors intended to establish K+ sensors with a higher dynamic range and stronger signals.*

We totally agree with the Reviewer’s comments that RGEPOs should be benchmarked against established genetically encoded potassium biosensors. Therefore, for comparison, we decided to use GINKO2, which is also a single-FP-based potassium biosensor characterized by the highest sensitivity among all other single-FP K-biosensors (doi:10.1371/journal.pbo.3001772). Moreover, due to its green fluorescence, GINKO2 can be co-imaged with RGEPOs in the same cell.

RGEPOs and GINKO2 were co-expressed in cultured neurons via calcium phosphate transfection, and dual-color imaging was performed 7 days post-transfection. Neurons were stimulated with 30 mM KCl, followed by perfusion with ACSF to wash out the elevated extracellular potassium. As a result, we observed that upon transient K⁺ stimulation, extracellular Asp-RGEPO1 exhibited a robust fluorescence increase of approximately 99%, while GINKO2 showed a fluorescence decrease of about 43% (Figure R3a,b). In contrast, intracellular RGEPO2 displayed a fluorescence decrease of approximately 51%, accompanied by around 50% decrease in GINKO2 fluorescence (Figure R3c,d). Importantly, the sensitivity and kinetics of the response for RGEPOs and GINKO2 were comparable. Moreover, these results demonstrated that RGEPOs can be co-imaged with the established green potassium sensor GINKO2 in primary neurons, enabling dual-color imaging and simultaneous monitoring of potassium dynamics.

**Figure R3.** Benchmarking of RGEPOs against GINKO2 in cultured mouse neurons. (a) Representative dual-color wide-field imaging of RGEPO1 and GINKO2 dynamics in dissociated hippocampal neurons in response to 30 mM KCl. (b) Time course of fluorescence intensity changes of RGEPO1 and GINKO2 with stimulation of 30 mM KCl in neurons (n = 51 neurons from 3 independent cultures). (c) Representative dual-color wide-field imaging of RGEPO2 and GINKO2 dynamics in dissociated hippocampal neurons in response to 30 mM KCl. (d) Time course of fluorescence intensity changes of RGEPO2 and GINKO2 with stimulation of 30 mM KCl in neurons (n = 22 neurons from 3 independent cultures).

These results demonstrated that the sensitivity and kinetics of RGEPOs are comparable to those of GINKO2. The newly obtained results were added as Supplementary Fig. 25 and described in the main text (please see lines 434-444 in the result section and lines 842-845 in the method section).

*2)      In general, the number of biological replicates is rather low for several experiments. Also, it is not always clear how many technical / biological replicates were included in the analysis. For example, the number of independent cultures is presently only n=1 or 2 in Fig. 5 and n=1 mouse was assessed for Fig. 7. It is important to increase the number of cultures / mice at least to n = 3. As for Figure 7, it is essential to corroborate the in vivo data with further replicates, otherwise it is not possible for the reviewer to assess whether the findings shown are coincidental or can be reproduced are therefore of biological significance.*

We thank Reviewer for pointing this out. To improve the reproducibility of the reported results, we have repeated those experiments, which had insufficient replicates in the original manuscript to obtain at least n of 3 independent experiments/cultures/mice. Specifically, for Figure 2i and l, we added two additional independent cultures for the EC_50_ measurements, resulting in a total of three independent cultures. For Figure 5b, we added two more independent cultures of primary neurons, bringing the total to three. For Figures 5d, f, i, and l, we also added two more independent cultures for EC_50_ determination, increasing the total to three independent cultures. For Figure 7, we recorded three mice for each sensor. In the case of RGEPO1, one mouse was excluded from analysis due to death before the spreading wave could be recorded, and thus, data from two mice were included in the final results. For RGEPO2, all three mice were successfully recorded and included in the analysis.

We have updated the relevant panels in the revised manuscript and included detailed descriptive statistics in the figure legends, clearly stating the total number of imaged cells, the number of independent experiments, and the number of replicates used. Additionally, please see the newly added Supplementary 9 (representative traces from more independent cultures in Figure 2), Supplementary Figure 24 and 27 (additional representative traces from more independent cultures shown in Figure 5), and Supplementary Figure 31 (increased replicates for mouse imaging in Figure 7).

We confirm that all results were reproducible within acceptable variability.

*3)      During optimization of the biosensors, the highest K+ concentration applied varied from 150 mM to 300 mM. Why?*

We thank Reviewer for this comment. During biosensor optimization, we used potassium concentrations ranging from 150 mM to 300 mM, depending on the system and purpose of each experiment. Specifically, 200-230 mM K⁺ was applied in the *E. coli* screening system to ensure a high signal dynamic range for ΔF/F-based selection. In mammalian cells, 200 mM K⁺ was used for a similar purpose, as this concentration slightly exceeds the intracellular physiological level. This strategy allows us to rapidly identify sensor variants with strong responsiveness under saturated conditions, which serves as an efficient first-pass screen. For the molecular dynamics simulations, 300 mM KCl was used along with 20 mM KCl to model RGEPO1 and RGEPO2 under both low and high potassium environments. The high concentration was intended to ensure that the binding sites were fully saturated. Importantly, the selected candidates were subsequently validated under physiologically relevant potassium concentrations to ensure their suitability for biological applications. We also confirm that the exact potassium concentrations used in each experiment have been clearly reported in the main text and corresponding figure legends.

*4)      Figure 2a and line 139-141: The authors choose to treat cells simultaneously with valinomycin, CCCP and 200 mM K+. Emptying of intracellular K+ by a K+ ionophore (f.ex. Gramicidin) would result in decreased intracellular K+ levels, following treatment with high K+ concentrations should result in steep increase of ΔF/F. This is something that should be tested to estimate the effect of the intracellular K+ ions on the sensors.*

We thank Reviewer for this very insightful suggestion. As noted, our original experiments used valinomycin and CCCP to equilibrate intracellular and extracellular K⁺ in HEK293FT cells, but this approach does not actively deplete intracellular K⁺. To better assess the impact of intracellular K⁺ levels on sensor performance, we conducted additional experiments using gramicidin, which facilitates K⁺ efflux and effectively reduces intracellular K⁺ concentration before external K⁺ is applied.

Consistent with the Reviewer’s suggestion, gramicidin-treated HEK293FT cells expressing RGEPO2 exhibited a significantly enhanced ΔF/F response of 334%, compared to 130% in cells treated with valinomycin and CCCP. The EC₅₀ also shifted from 16.8 mM (valinomycin + CCCP) to 43.3 mM (gramicidin; Figure R4). These results demonstrated that a lower intracellular K⁺ baseline leads to a steeper fluorescence increase upon external K⁺ stimulation, resulting in an EC_50_ value closely matching that measured in solution, where we can precisely control [K^+^].

These results confirmed that RGEPO2 responded to intracellular K⁺ dynamics and that ionophores and external K⁺ gradients serve as effective experimental tools to modulate intracellular K⁺ levels. We appreciate the reviewer’s suggestion, which enabled us to gain a more mechanistic understanding of how intracellular K⁺ levels shape sensor output. The new data have been added to the revised manuscript (please see Fig. 2j,k,l and revised Table 1) and described in the main text (see lines 188-194 in the results section, lines 799-801 in the methods section).

**Figure R4.** (a) Representative images of expression and fluorescence change of RGEPO2 in response to [K^+^]=200 mM. Scale bar, 10 µm. (b) Plot of normalized ∆F/F against different K^+^ concentrations fitted using nonlinear fitting (Hill) for the data shown in panels c, d, and e (n = 97 cells from 3 independent cultures). (c–e) Time courses of fluorescence intensity change (ΔF/F) of RGEPO2 in HEK293FT cells stimulated with a series of K⁺ buffers in the presence of 10 μg/mL gramicidin. Each trace represents data from an independent culture (n = 29, 32, and 36 cells, respectively). Data are shown as mean ± SD.

*5)      Line 180 and Supplementary Figure 8a: In the introduction, the extracellular concentration of K+ was mentioned (3.5 to 5 mM). However, during the perfusion experiment, the authors used higher extracellular K+ concentrations (7-9 mM). Why? How does the physiological K+ range of 3-5 mM alter the fluorescence signals? Additionally, the authors state that the fluorescence signals return to baseline levels. The ΔF/F0 intensity curve, however, seems to decline even below baseline levels. How is this explained?*

We thank Reviewer for pointing out this inconsistency. The original experiments in Supplementary Figure 8a were designed to assess the reversibility of sensor fluorescence responses in mammalian cells, and the use of 7–9 mM K⁺ aimed to elicit a robust ΔF/F signal. However, we agree that testing the sensor within the physiological range (3.5–5 mM K⁺) is important for biological relevance.

To address this concern, we repeated the experiments using additional extracellular K⁺ concentrations of 2 mM and 8 mM, and observed robust ΔF/F responses of ~80% and ~140%, respectively. These new data confirm that RGEPO1 responds reliably within and beyond the physiological range of K⁺. The updated results are shown in the revised Figure 2h and Supplementary Figure 9a,b, and referenced in the main text (please see lines 182-185 in the results section). Please see the newly generated results in Figure R5 below.

**Figure R5.** (a-c) Time courses of fluorescence intensity change (ΔF/F) of extracellular Asp-RGEPO1 on HEK293FT cells stimulated with a series of K⁺ buffers. Each trace represents data from an independent culture (n = 27, 33, and 25 cells, respectively).

Regarding Reviewer’s observation that the fluorescence signal appears to fall below baseline, we believe this effect is primarily due to photobleaching. Please note that the duration of many recordings exceeded 10 min, and the presented traces were not corrected for photobleaching.

*6)      Line 181-182 and Figure 2d, g, h: Representative images in Figure 2d and Figure 2g show the membrane-located RGEPO1 at 0 mM and 20 mM K+. In Figure 2h, the maximal K+ concentration plotted corresponds to 50 mM and the change in ΔF/F was calculated to 286 % for 50 mM. It would be more consistent if representative images, curves and calculations are comparable within one figure.
Similarly, in line 384 and Figure 5h, the maximal K+ concentration applied is 20 mM and ΔF/F calculations were performed for this concentration. Supplementary Movie 5, however, presents the addition of 50 mM KCl. Please revise to show the same maximal concentration for the representative images, movies and calculations.*

Thank you for pointing these out. We revised Figure 2g to show cells at the maximal tested concentration of 50 mM. Please see revised Figure 2g in the main text.

Correspondingly, we revised Figure 5h to show the same maximal concentration for the representative images, movies, and calculations. Please note that we provided new images and extended the original datasets with new experimental data to increase the number of independent experiments as requested in point 2 above.

*7)      Line 203ff: RGEPO1 was characterized in solution without the ASP leader or PDGFβ anchor. In the experimental set-up, especially in the in vitro and in vivo experiments, RGEPO1 was used with the ASP leader and PDGFβ anchor to target the plasma membrane of the cells. The missing biophysical characterizations of the RGEPO1 fusion protein must be provided.*

The leading peptide and tether were added for mammalian expression only. The RGEPO1 fusion with these peptides is not properly expressed in *E. coli*. Therefore, we performed a systematic and comprehensive characterization of Asp-RGEPO1 in mammalian cells, including HEK293FT cells, neurons, and astrocytes. The addition of these localization motifs should not influence the spectral properties of RGEPO1 measured in solution. Moreover, Asp-RGEPO1 is intended only for expression in mammalian cells.

*8)      Line 230-231 and Figure 3e: The authors described the ion selectivity of RGEPO1 and RGEPO2, which is important and highly appreciated. However, the data shows an influence of Ca2+ on RGEPO fluorescence intensities. As K+ and Ca2+ play an important role in cellular ion homeostasis and influence each other, the performed molecular dynamic simulations should also examine the Ca2+ binding sites in the sensors. Along these lines it is unclear whether and how parallel analysis of RGEPO and GCaMP6f signals affect each other during multiplexing. Hence, some representative experiments (e.g. 5a-d) must be reproduced with RGEPO (without GCaMP6f) and vice versa with GCaMP6f (in the absence of RGEPO).*

This is an important point. In Figure 2e, the used concentration for calcium is 20 mM, which is 20 times higher than the maximal physiologic [Ca^2+^] in the cytoplasm of neurons. In the revised manuscript, we performed an additional in vitro titration of RGEPO1 and RGEPO2 with Ca^2+^ in the range from 0 to 1.1 mM matching physiological [Ca^2+^] in neurons (under resting conditions, neurons actively maintain a steep gradient between low intracellular free Ca^2+^ concentration ([Ca^2+^]_i_=0.1–0.5μM) and high extracellular Ca^2+^ levels ([Ca^2+^]_i_∼1 mM) achieved during action potentials (doi: 10.3389/fphar.2012.00061)). Correspondingly, we conducted a series of calcium titrations with a maximum concentration of 1.1 mM, demonstrating that both RGEPOs do not respond to Ca²⁺ under physiological conditions (see Figure R6 below, which is added as new Supplementary Fig. 14 to the revised manuscript).

**Figure R6.** Ca^2+^ titration of RGEPOs in the range of 0 to 1.07 mM in solution.

In addition, MD simulations further confirmed the ion selectivity of RGEPOs, as Ca²⁺ binding was found to be transient and insufficient to induce the conformational changes required for fluorescence response. These results further confirm the high specificity of the RGEPO sensors. We revised the main text to describe new results and methods (please see lines 260-264 and 327-342 in results section, lines 607-609 in discussion, and lines 736-738 in the methods section) and added new Supplementary Fig. 14 with experimental results and Supplementary Fig. 17 with MD simulation results.

*9)      Figure 5c and Figure 5e: In both panels, the expression of RGEPO2 in neurons is presented. However, the expression pattern appears to be different as in Figure 5c, RGEPO2 is expressed in the cytoplasm and nucleus of the neurons (soma and dendrites) whereas in Figure 5e, the expression is mainly restricted to the nucleus (?) and absent in the dendrites. Please clarify!*

Thank you for bringing it up. Figure 5c shows a maximum intensity projection confocal image, which allows for clear visualization of neuronal projections. In turn, Figure 5e shows a wide-field image of the neurons, which, due to a focus on cell bodies (since we recorded major fluorescence signal from cell bodies), does not allow for very clear viewing of dendrites. We have revised the figure legend to specify imaging modalities for each present image in the figure.

*10)     Line 362-365 and Figure 5d: Glutamate stimulation provoked a K+ efflux in neurons. The Glutamate-provoked Ca2+ dynamics should be shown. Also, the glutamate-induced K+ efflux has already been studied by others using the FRET-based K+ sensor GEPII and this should be fairly mentioned in the MS (doi: 10.1096/fj.202002308RR).*

We thank Reviewer for this valuable suggestion. To address this point, we performed additional experiments in which RGEPO2 and GCaMP6f were co-expressed in cultured neurons to simultaneously monitor glutamate-induced K⁺ and Ca²⁺ dynamics. Upon transient glutamate stimulation, GCaMP6f showed a robust fluorescence increase of approximately 1076%, while RGEPO2 exhibited a fluorescence decrease of about 48%, indicating intracellular K⁺ efflux (see Figure R7 below). These results are consistent with previous findings using the FRET-based potassium sensor GEPII, which demonstrated a pronounced intracellular K⁺ decrease upon NMDA receptor activation in primary cerebellar granule neurons (10.1096/fj.202002308RR).

**Figure R7.** (a) Representative images of cultured neurons co-expressing RGEPO2 and GCaMP6f in ACSF buffer before and after stimulation with 500 μM glutamate. Scale bar, 10 µm. (b) Time course of fluorescence intensity changes of RGEPO2 with stimulation of 500 μM Glutamate (n = 91 neurons from 3 independent cultures).

We revised the main text to cite previous work by Ehinger et al. and added the new results (please see lines 413-421 in the result section) and the corresponding data to Figure 5c and d.

*11)     Line 368-376 and Figure 5e: As Ca2+ may directly affect the fluorescence signals of the RGEPO biosensors, it is necessary to chelate Ca2+ and to represent the K+ dynamics recorded by RGEPO2 under these conditions. Additionally, the authors state that membrane depolarization occurs in response to the K+ stimulus. Has this statement been validated by using e.g. voltage-sensitive dyes?*

We thank Reviewer for this insightful comment. As addressed in our response to point 8, we performed in vitro calcium titrations of RGEPO1 and RGEPO2 over a range of 0 to 1.07 mM Ca²⁺. These experiments demonstrated that both sensors exhibit negligible fluorescence changes across the physiological Ca²⁺ range (0.1–1.07 mM), indicating that Ca²⁺ does not interfere with RGEPO signals under physiological conditions (see Figure R6 above).

Regarding the membrane depolarization statement, we acknowledge that we did not perform voltage-sensitive dye imaging in this study. However, the relationship between elevated extracellular K⁺ and membrane depolarization is well established. Increasing extracellular K⁺ reduces the transmembrane K⁺ gradient, resulting in membrane depolarization and subsequent activation of voltage-gated Ca²⁺ channels, which leads to Ca²⁺ influx. This is a widely documented physiological response in excitable cells, including neurons, as demonstrated in previous studies (dio:10.1177/1759091420974807; [10.1523/JNEUROSCI.18-03-01047.1998](https://doi.org/10.1523/jneurosci.18-03-01047.1998)).

*12)     Line 381 and Supplementary Figure 17: Please confirm the membrane localization of ASP-RGEPO1 in astrocytes by a plasma membrane counterstain. This is important as in the respective figure, astrocytes seem to express membrane-located RGEPO1 in the cytoplasm.*

Thank you for raising this point. To confirm the plasma membrane localization of Asp-RGEPO1 in astrocytes, we co-expressed RGEPO1 with the membrane-targeted EGFP marker Igκ-EGFP. Confocal fluorescence imaging and normalized linecut analysis of fluorescence intensity across the cell profile revealed strong colocalization between RGEPO1 and Igκ-EGFP, supporting reliable membrane localization of Asp-RGEPO1 in astrocytes (see Figure R8 below, also added to the revised manuscript as new Supplementary Figure 26).

**Figure R8.** Fluorescence images and membrane localization analysis of Asp-RGEPO1 expressed on the extracellular surface of astrocytes. Membrane-targeted EGFP (Igκ-EGFP) was co-expressed to label the plasma membrane. Left, single-plane confocal fluorescence images of astrocytes expressing the RGEPO1 (red) and EGFP (green) (n= 4 cells from one independent culture). Right, normalized linecut (shown as white dashed line on the left) plots of fluorescence signals measured in both the red and green channels. Scale bars, 20 µm.

*13)     Line 413 and Supplementary Figure 20, 21: The expression of RGEPOs is mainly recognized in the right hemisphere even if the authors mentioned in the methods part that the injection was performed in both hemispheres. Is there any explanation for this?*

We thank Reviewer for this insightful comment. As described in the Methods section, AAV injections were performed bilaterally. In Supplementary Figure 29 (Asp‑RGEPO1), the expression appears on both hemispheres. In Supplementary Figure 30 (RGEPO2), the right hemisphere shows stronger fluorescence, while the left side displays detectable expression, but much weaker than the right hemisphere.

This asymmetry may arise from technical variability during manual injection, such as slight differences in pipette placement, angle, or tissue backflow, which can influence viral distribution. In addition, the selected brain section may have included more of the expression region in the right hemisphere, as differences in sectioning depth could also contribute to apparent asymmetry. These technical differences are commonly encountered in intracerebral viral delivery and do not affect our main conclusions. We have now clarified this point in the legend of Supplementary Fig. 30.

*minor*
*1.)     The authors have to exclude several spelling mistakes in the manuscript. As an example:*

*line 128: performance;*

Corrected.

*Line 179: RGEPO;*

Corrected.

*Figure 6f: RGEPO2 is indicated in the x-axis even if the experiment was performed with RGEPO1;*

Fixed.

*Line 354, Figure 5a: GcaMP6f is not highlighted in the panel;*

We added details in the figure legends.

*Line 1189* *and Supplementary Figure Legend 8c RGEPO1 described instead of RGEPO2. Please correct!*

Corrected.

We really thank Reviewer for pointing out these typos and mistakes. All noted issues have been carefully addressed in the revised manuscript. Specifically, we corrected the typographical and labeling errors, including “performance” (line 128), “RGEPO” (line 179), corrected the label in Figure 6f to indicate RGEPO1, highlighted GCaMP6f in Figure 5a, and revised the mislabeling of RGEPO1 to RGEPO2 in Supplementary Figure Legend 8c.

*2.)     The terminology used for the HEK cells is inconsistent throughout the manuscript. The cells are either described by HEK293FT, HEK or HEK293T. As different variants of HEK293 cells are used in research, please specify the variant in the whole manuscript or introduce an abbreviation.*

We thank Reviewer for pointing this out. It is important to be specific. In the manuscript, we used only HEK293FT cells. The terminology for HEK cells has been standardized to "HEK293FT" throughout the manuscript.

*3.)     The effects of kainic acid on hippocampal neurons have recently been monitored using the GEPII K+ biosensor (10.1038/s42003-023-05387-9) in vitro. This is something that may be interesting for the discussion of the present study.*

We appreciate the Reviewer’s suggestion regarding the recent study using the GEPII sensor to monitor kainic acid-induced K⁺ dynamics in hippocampal neurons (DOI: 10.1038/s42003-023-05387-9). We have cited this paper in the revised manuscript. This study reported an intracellular potassium decrease in vitro using GEPII. In our in vivo experiments, RGEPO2 similarly revealed a decrease in intracellular potassium during kainic acid-induced seizures in awake mice. Additionally, RGEPO1 showed an increase in extracellular potassium levels under comparable conditions. Including this reference helps illustrate how our in vivo findings align with prior in vitro observations and highlights the broader applicability of RGEPO sensors for monitoring seizure-associated potassium dynamics.
