# Supplementary material for: Sensitive red fluorescent indicators for real-time visualization of potassium ion dynamics in vivo
Source: PLoS Biol. 2025 Sep 17;23(9):e3002993. doi: 10.1371/journal.pbio.3002993 (PMC12456824; doi:10.1371/journal.pbio.3002993)
Supplement: Attachment — Submitted filename: Response to reviewers.docx [file pbio.3002993.s048.docx]

Dear Richard,

Thank you for your support and assistance throughout the revision of this manuscript. Enclosed, please find the point-by-point response to the reviewers' comments.

*Reviewer remarks:*

*Reviewer#1 (Yulong Li, identifies himself): In the revised submission, the authors addressed all of my concerns and suggestions. In response to many valuable comments from the Reviewer 2, the authors further improved the manuscript and convincingly demonstrated that RGEPOs are excellent tools for studying K+ dynamics both in vitro and in vivo. I believe the manuscript is now ready for publication.*

We are grateful to Reviewer for the valuable and insightful comments and, of course, for the overall very positive feedback on our new genetically encoded potassium biosensors.

*Minor point: "RGPO2Cin" in the legend of newly added Supplementary Figure 13 should be "RGEPO2 in".*

We have revised the legend of Supplementary Figure 13 to correct the typo.

*Reviewer#2: The authors have answered to all my concerns and suggestions appropriately. The manuscript as greatly improved.*

We express our sincere gratitude for the critical and insightful comments, which have significantly contributed to enhancing our manuscript. We truly appreciate the time and effort Reviewer has dedicated to aiding us with the valuable feedback on the revisions.
